# Multi-Targeted Metabolic Profiling of Carotenoids, Phenolic Compounds and Primary Metabolites in Goji (*Lycium* spp.) Berry and Tomato (*Solanum lycopersicum*) Reveals Inter and Intra Genus Biomarkers

**DOI:** 10.3390/metabo10100422

**Published:** 2020-10-21

**Authors:** Doriane Dumont, Giorgia Danielato, Annie Chastellier, Laurence Hibrand Saint Oyant, Anne-Laure Fanciullino, Raphaël Lugan

**Affiliations:** 1 Institut National de la Recherche Agronomique, Plantes et Systèmes de Culture Horticole, 228 Route de l’aérodrome, Domaine Saint Paul, Site Agroparc, CS 40509, 84914 Avignon, France; doriane.dumont@inrae.fr; 2 Unité Mixte de Recherche QualiSud, Campus Jean Henri Fabre, Avignon Université, 301 rue Baruch de Spinoza, BP21239, 84916 Avignon, France; giorgia.danielato@gmail.com; 3 IRHS-UMR1345, Université d’Angers, INRAE, Institut Agro, SFR 4207 QuaSaV, 49070 Beaucouzé, France; annie.chastellier@inrae.fr (A.C.); laurence.hibrand-saint-oyant@inrae.fr (L.H.S.O.)

**Keywords:** *Lycium barbarum*, *Lycium chinense*, *Solanum lycopersicum*, UPLC–MS, GC–MS, carotenoid extraction

## Abstract

Metabolic profile is a key component of fruit quality, which is a challenge to study due to great compound diversity, especially in species with high nutritional value. This study presents optimized analytical methods for metabolic profiling in the fruits of three *Solanaceae* species: *Lycium barbarum*, *Lycium chinense* and *Solanum* *lycopersicum*. It includes the most important chemical classes involved in nutrition and taste, i.e., carotenoids, phenolic compounds and primary compounds. Emphasis has been placed on the systematic achievement of good extraction yields, sample stability, and high response linearity using common LC-ESI-TQ-MS and GC-EI-MS apparatuses. A set of 13 carotenoids, 46 phenolic compounds and 67 primary compounds were profiled in fruit samples. Chemometrics revealed metabolic markers discriminating *Lycium* and *Solanum* fruits but also *Lycium barbarum* and *Lycium chinense* fruits and the effect of the crop environment. Typical tomato markers were found to be lycopene, carotene, glutamate and GABA, while lycibarbarphenylpropanoids and zeaxanthin esters characterized goji (*Lycium* spp.) fruits. Among the compounds discriminating the *Lycium* species, reported here for the first time to our knowledge, chlorogenic acids, asparagine and quinic acid were more abundant in *Lycium chinense*, whereas *Lycium barbarum* accumulated more lycibarbarphenylpropanoids A-B, coumaric acid, fructose and glucose.

## 1. Introduction

Fruits and vegetables are important components of the human diet. Their consumption is associated with beneficial health effects, partly due to their high contents of phytomicronutrients such as carotenoids and phenolic compounds [1]. Goji berry, labeled a “superfood” because of its high content in phytomicronutrients, belongs to the *Lycium* genus [2] in the Solanaceae family, like tomato, which is one of the most important food commodity in the world. Tomato is an important model species for the biology of fleshy fruits and nutritional studies, and hence its genetic proximity with *Lycium* makes it a relevant reference of which to compare to goji. The complex composition of goji and tomato fruits has been the subject of several studies, using various metabolomics approaches to address the inherent challenges of profiling of a very high number of compounds with a vast array of physico-chemical properties.

Modern metabolomics relies on two main approaches, namely non-targeted and multi-targeted. In non-targeted metabolomics, the experiments are performed without a hypothesis on specific metabolites; consequently, the samples are extracted with a mixture of solvents able to recover a large range of metabolites in a particular range of polarity; subsequently, high resolution mass spectrometry data are usually acquired after a reverse phase chromatographic separation. Dedicated to high throughput and semi-quantitative exploratory analysis, it is very efficient at profiling dozens of metabolites with quite similar physicochemical properties. The main limitation is the difficult annotation of metabolites, especially in plants, because mass spectra databases are far from comprehensive, and mass spectra are method/apparatus dependent. A good example of non-targeted analysis is provided by Gomez-Romero et al. [3] who isolated 135 metabolites belonging to various chemical classes in tomato cultivars using HPLC–ESI–TOF–MS. The method was validated for the quantification of 11 phenolic compounds and, interestingly, 21 metabolites were reported in tomato for the first time. Other non-targeted LC–MS implementations were given by Bondia-Pons et al. [4] and Lv et al. [5], who found biomarkers of geographic origin of goji berries, but also pesticide traces and putative markers of medicinal material adulteration. Multi-targeted profiling is a more established approach that consists of specific procedures to analyze a particular compound class. Its efficiency has greatly improved in the last decades with mass spectrometry-based detection systems such as triple quadrupoles, which are more versatile than diode array detectors (DAD) and provide a more effective third dimension for peak integration. The main advantages are faster and more reliable identification and quantification, while the main disadvantage is the inability to discover new molecules, the analysis being by definition circumscribed to a set of a priori selected compounds. Goji fruits have been screened for carotenoids to highlight differences between cultivars, the impact of harvesting periods or climatic characteristics of cultivation areas [6,7]. More widely multi-targeted analysis has been performed by Zhang et al. [8] on carotenoids, total polysaccharides and a dozen of phenolic compounds in various genetic backgrounds. Recently, Wang et al. [9] profiled many primary metabolites, phenolic compounds and also some phytohormones to understand the climatic factors influencing the goji fruit morphology and composition. An important issue for metabolomics is the coverage of metabolites, which is ideally comprehensive. A single extraction and analysis procedure is unable to provide information on different compound classes, e.g., untargeted LC–MS profiling of medium polarity extracts is limited due the poor retention and separation of more polar compounds, such as osides, amino acids and organic acids analysis that demand the development of dedicated LC–MS or GC–MS methods, not to mention the quasi absence of non-polar compounds in such extracts, which would require a dedicated extraction procedure. Remarkably, neither the untargeted nor the multi-targeted studies of Wang et al. [9] mentioned above encompassed non-polar metabolites such as carotenoids. This can be overcome by combining multiple extractions and analytic designs to cover more chemical classes in the samples.

Highly and often specifically influenced by the genetic background and the environment, the metabolic phenotype is a consistent -omic level to study the stress resistance mechanisms that determine crop management and varietal improvement. Since fruit metabolic profiles are significantly impacted by climatic conditions and cultural practices, inter and intra genus comparisons cannot rely on existing data but instead require dedicated studies with homogenous cultivation conditions, as presented in this study [10,11]. Metabolomics is also of great interest for food qualification such as molecular-based traceability and nutritional value. Genetic certification by markers is of particular importance, since two species, *Lycium barbarum* (Lb) and *Lycium chinense* (Lc), are referred to as goji plants. They present similar red ovoid fruits and are difficult to discriminate with morphological or molecular markers [12,13]. Goji berry is widely used for its nutritional properties in Asia [2] and has been imported in Europe for several decades in its dried form [12]. The genetic origin of commercial cultivars being difficult to establish, some mixes of Lb and Lc berries are commercialized as food or food ingredients, although only the fruits of Lb are authorized in Europe, since they have been consumed in Europe before 1997 and are not under the novel food legislation [12,14]. Recently, a molecular method based on the psbAtrnH barcoding marker allowed for the discrimination of Lb from Lc [14], but to our knowledge no metabolic marker has been reported so far.

The objective of this paper was to develop high-throughput methods, from metabolite extraction to data mining, for multi-targeted metabolic profiling amenable to biomarker discovery in goji berry. The following metabolite families were chosen for their importance in stress responses and fruit quality in terms of taste and nutrition: carotenoids, phenolic compounds and primary metabolites such as simple sugars, amino acids and organic acids. The literature contains a significant number of techniques for the determination of carotenoids. In complex matrices such as goji berry, most techniques use C30 columns and a strong methyl tert-butyl ether (MTBE)-based solvent [15]. These operating conditions require a relatively long run and the need for a chromatographic system compatible with MTBE. Our goal was also to develop a method free from saponification, with a relatively “cheap” run, as well as short time, common C18 column, electrospray ionization and elution solvents compatible with conventional UPLC chromatographic system. The profiling of phenolic compounds was less challenging, since these compounds are easily amenable to relatively short and standard UPLC–ESI–MS/MS runs. Finally, primary metabolites were analyzed with a well-established GC–EI–TOF–MS technique that provides a good coverage of small polar molecules and high reliability in terms of both peak annotation and quantification [16,17].

Method development focused on the quantitative aspects of extraction and analysis to insure efficient sample comparison. Extraction yields, sample stability, dose/response linearity and matrix effect were thus systematically tested, and the methods proved to be able to discriminate the *Lycium* and *Solanum* genera, the two *Lycium* species, Lb and Lc, and also factors with more subtle impact on metabolic profiles such as the year of harvest and the sampling methods.

## 2. Results and Discussion

### 2.1. Carotenoid Profiling Optimization and Validation

#### 2.1.1. Extraction

**Figure 1:**
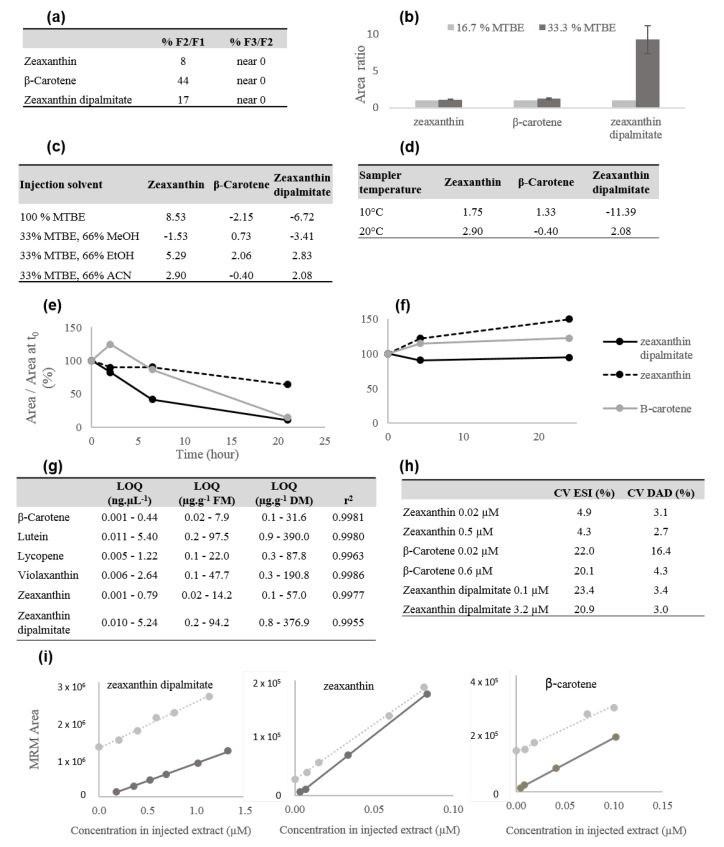
Carotenoid extraction optimization and quantitative analysis validation. (**a**) Determination of the minimum number of successive extractions to recover the carotenoids; 3 fractions (F1–F3) were obtained, and the Multiple Reaction Monitoring (MRM) peak areas ratios were reported. (**b**) Impact of the MTBE percentage in the injection solvent on the MRM signal intensities. (**c**) Impact of 4 different injection solvents on the variation rate of the MRM peak area over 4 h, expressed as the percentage of variation per hour. (**d**) Auto-sampler temperature effect on the carotenoid stability, expressed as the percentage of MRM peak area variation per hour. (**e**,**f**) Carotenoid stability estimated from the variations of signal intensities over time before (**e**) and after (**f**) method optimization. (**g**) Limit of quantification (LOQ) expressed as ng.µL^–1^ of extract and µg.g^–1^ of fresh or dry weight; the response linearity r^2^ was calculated from the standard mixture at a minimum of 3 concentration levels. (**h**) Precision of the method estimated from the ESI or DAD peak areas coefficient of variation (%) from 9 injections over 2 days, given for 2 concentrations. (**i**) Evaluation of the matrix effect on MRM peak areas, dose/response curves of pure standard (black) and standard added to goji extract (grey).

Carotenoid profiling in goji fruits is challenging because they display very contrasted concentrations and chemical properties affecting their solubility and stability during the extraction process. The best solvent mixture was found to be EtOH:hexane followed by the addition of water and hexane as already described by Fu et al. [18]. The mixtures hexane:acetone:EtOH:water detailed by Aubert et al. [19] and MeOH:ethyl acetate:ether petroleum described by Hempel et al. [20] resulted in lower signal intensities for both free and esterified xanthophylls (e.g., 30% and 50% less signal for zeaxanthin and zeaxanthin dipalmitate, data not shown). In addition we determined that a fresh sample to solvent volume ratio greater than 150 mg/mL resulted in solvent saturation; thus, the final ratio selected for this study was 80 mg/mL, in agreement with Hempel et al. [20]. The addition of 30 mg of CaCO_3_ did not significantly affect the stability of the molecules, in accordance with Mercadante et al. [15], but as a precautionary measure, the addition of CaCO_3_ in the extraction phase was retained to eliminate a potential risk of carotenoid rearrangement [21]. Conversely the addition of 0.1% of BHT, a lipophilic antioxidant commonly used in the food industry (E321) improved the compound’s stability over time (data not shown). To insure the exhaustion of the plant material, successive extractions of the pellet were performed, and it appeared that two successive extractions were required and sufficient to fully recover the carotenoids (Figure 1a). Since the extraction solvent is not suitable for chromatographic injection due to the presence of hexane, the extracts were dried and recovered in different solvent mixtures (injection solvent). The injection solvent needed to both insure a maximum dissolution of the compounds and be compatible with the chromatographic system. Gupta et al. [22] indicated that a minimum amount of MTBE is required, and we found that a mixture of EtOH:MTBE (2:1 *v*/*v*) was associated with a high increase in the signal of zeaxanthin dipalmitate, the most apolar carotenoid found in our sample (Figure 1b); it also provided satisfying compound stability (Figure 1c). This effect is linked to the lower polarity index of MTBE compared to ethanol (2.5 and 5.8 respectively), which greatly improves the solubilization of the less polar carotenoids. Another unexpected but critical parameter regarding carotenoid stability was found to be the auto-sampler temperature; the signal associated with zeaxanthin dipalmitate decreased at the rate of 11% per hour when the sample was kept at 10 °C, probably due to crystallization, while it was stable at 20 °C (Figure 1d). The whole extraction/conservation process was validated by checking the carotenoid stability over 25 h after extraction (Figure 1e,f).

#### 2.1.2. UPLC–MS

Given the apolar nature of carotenoids, chromatographic separation is usually performed on C30 reverse phase columns with organic solvent such as MTBE, while the detection of these molecules by mass spectrometry involves mostly Atmospheric Pressure Chemical Ionization (APCI) [8,20,22,23,24]. However, other techniques were reported using more standard devices. Pop et al. [25] proposed a reverse phase separation and an ESI ionization of free and esterified xanthophylls. We thus developed the separation of carotenoids on a C18 column and a quantification method based on ESI in positive mode, with xanthophylls producing protonated cations [M+H]^+^ while less polar carotenoids such as carotene producing radical cations [M]^+^. The column temperature was tested between 30 and 55 °C, showing a better selectivity without signal loss at 55 °C, in good agreement with de Quiros and Costa [23] (Figure S1).

Six major carotenoids (compounds **1**, **2**, **4**, **7**, **8** and **11** in Table S1) were identified with a level 1 confidence by comparing their retention time, absorbance spectra and MS/MS data with authentic standards [26] (Figure S2). Moreover, five carotenoids were identified with a level 2 confidence by comparing their MS/MS and DAD spectra with published data (Table S1). Compound **3** showed the following spectral signatures consistent with phytoene: absorbance peaks at 275, 286 and 298 nm and *m/z* 545.41 [15,20,27]. Compounds **5** and **6** were putatively identified as neochrome or luteoxanthin with main absorbance at 399, 424, 450 and 401, 424 and 450 nm respectively [28] and a major ion at *m/z* 601.32. Compound **9** was assigned to zeaxanthin palmitate with main absorbance at 429, 453 and 479 nm and *m/z* 807.4 [M+H]^+^, producing the expected fragment ions *m/z* 789 [M+H-H_2_O]^+^, *m/z* 551 [M+H-palmitic acid]^+^ and *m/z* 553 [M+H-H_2_O-palmitic acid]^+^ [15,20,27]. Compound **10** showed the absorbance spectra of auroxanthin (380, 400 and 425 nm) [29] and a major ion [M+H]^+^ at *m/z* 811.7 corresponding to auroxanthin myristate, as well as the fragment ion *m/z* 565 characteristic of esterified forms of auroxanthin [30]. Two putative carotenoids were also detected (level 3 confidence) for which no published information was available, but their absorbance spectra matched the common carotenoid spectrum, absorbing at 405, 429, 455 nm and 424, 447 and 474 nm respectively. Overall, xanthophylls were the major class of carotenoids found in goji, especially zeaxanthin, either free or esterified with one or two palmitic acids, zeaxanthin dipalmitate being by far the most abundant, its absorbance spectra representing roughly 75% of the total carotenoids absorbance (Figure S3).

#### 2.1.3. Quantification

The LOQ and linearity of the method were assessed by injecting standard mixtures at concentrations ranging from 0.02 to 6.6 µM. The LOQ varied between 0.02 and 0.2 µg.g^–1^ Fresh Weight (FW) and the correlation coefficients were greater than 0.99 (Figure 1g), which is in the same order as in Protti et al. [31]. The precision (coefficient of variation) was calculated by injecting a mixture of standards during two consecutive days (nine injections). Figure 1h shows that coefficients of variation did not exceed 15% except for the β-carotene in DAD (16.4%), β-carotene and zeaxanthin dipalmitate MRM signals (up to 23.4%). Finally, the matrix effect was tested by spiking different solutions of carotenoids in sample extracts or pure solvent; the collinearity observed between the dose response curves indicates the absence of any significant matrix effect (Figure 1i).

### 2.2. Phenolic Compound Profiling Optimization and Validation

#### 2.2.1. UPLC–MS

Regarding chromatographic separation, Protti et al. [31] advocate a C18 column rather than a PFP column that obtains poor peak shapes. However, the F5 column is a new generation PFP column with core–shell technology, providing better separation efficiency to be achieved in a short time and without high pressure [32]. This column is involved in several interactions including aromatic interactions when MeOH is used as a strong solvent. These interactions increased aromatic compounds and peak resolution in our study as compared with the C18 column. Phenolics were better ionized in negative mode due to their acidic nature in agreements with Lin et al. and Goupy et al. [32,33].

Overall, the main categories of phenolic compounds found in this study were flavonol derivatives (15 out of 46 compounds) and lycibarbarphenylpropanoids (13 compounds) (Table S1). Twenty-three compounds were identified with a level 1 confidence [26] by comparing their retention time, absorbance spectra and MS/MS data with authentic standards. Twenty-three phenolic compounds were also identified with a level 2 confidence by comparing their MS/MS and DAD spectra with published data (Table S1). Compounds **16**, **17** and **19** were identified as isorhamnetin 3-O-rutinoside isomers (*m/z* 623 [M−H]^−^ and fragment *m/z* 577.2), this molecule has already been reported in goji fruits by Inbaraj et al. and Rocchetti et al. [34,35]. Compound **18** produced the ion *m/z* 771 characteristic of quercetin-rhamno-dihexoside, as already described by Inbaraj et al. [34], while the fragment *m/z* 301 was consistent with a quercetin moiety; 4-hydroxybenzoic acid was associated with compounds **32** and **33**, due to an absorbance peak at 260 nm and specific fragment ions *m/z* 137 and *m/z* 92.4 [36]. Recently Pedro et al. and Rocchetti et al. [35,37] also identified several isomers of hydroxybenzoic acid in goji fruits. Seven compounds (**43**–**45**, **48**, **50**, **51**, **53**) showed the MRM transition 487.2 > 162.69 and have already been described by Zhou et al. [38] as lycibarbarphenylpropanoid A or B related compounds. Compounds **44** and **47** were as assigned to lycibarbarphenylpropanoid E (*m/z* 503.18), whereas compounds **49**, **52**, **54** and **55** were assigned to lycibarbarphenylpropanoid C or D (*m/z* = 517.26) [38]. Finally, compounds **56** to **59** displayed the MRM transition 312.1 > 147.66 characteristic of N-feruloyl tyramine isomers [39].

#### 2.2.2. Quantification

A critical parameter of efficient quantitation is the extraction yield. Three ratios of fruit mass to solvent volume were tested, showing that a 1:30 ratio allowed the extraction of at least 93% in a single step for the three phenolic compounds tested (Figure S4a), in agreement with Pedro et al. [37]. The extraction was thus carried out in a single step by the addition of 1.8 mL of extraction solvent to 30 mg of fruit powder. The LOQ calculated for 17 compounds ranged from 0.17 to 0.94 ng.µL^−1^ (Figure S4b). Even though Protti et al. [31] found a much lower LOQ (for example 0.001 ng.µL^−1^ for chlorogenic acid, versus 0.35 in this paper), our purpose was not to quantify the phenolic compounds but to check the response linearity in a concentration range consistent with the amounts found in goji berries. The calibration curve correlation coefficients (r²) were high, between 0.87 and 0.99 (Figure S4b). As previously detailed for carotenoids, the linearity was also directly determined in goji extracts at various dilutions rates (from 0.5 to 100%). The 34 phenolic compounds analyzed displayed r² from 0.92 to 0.99 with a mean at 0.98 (data not shown). The precision of UPLC–TQ analysis, reflecting the combined variations in injection, ionization, detection and molecule stability was determined by injecting a series of standard mixtures at 5 µM (*n* = 9) over 3.5 days. The coefficients of variation were all found below 21%, with an average of 17.6% (Figure S4c), which is slightly higher than the value of 10% reported by Protti et al. [31]. Finally, as for carotenoids, no matrix effect was detected (Figure S4d).

### 2.3. Primary Metabolite Profiling Optimization and Validation

The fruit mass to solvent volume ratio was optimized to 3 mg/mL of solvent extraction. The linearity of the calibration curves was determined on pure standards from 0.5 to 100 µM; the corresponding correlation coefficients ranged from 0.98 to 1.00 (Table S2), whereas they ranged from 0.82 to 1.00 (mean 0.98) when they were calculated from goji extracts injected at 6 dilution rates. The whole sample preparation method, including vacuum drying, derivatization and injection, showed a response coefficient of variations less than 6.2% over 15 replicates (Table S3).

The goji fruit at maturity was reported to contain high levels of fructose, glucose, sucrose, citric acid, tartaric acid and quinic acid [40], which is consistent with our data, except for tartaric acid, which was not detected. Among the primary metabolites, carbohydrates/polyols and amino acids represented, respectively, 42% and 27% of the detected compounds (Table S4). Thirty-four primary metabolites were identified with a level 1 confidence [26] by comparing their retention time and EI–MS spectra with authentic standards. Among them, the most abundant were carbohydrates, organic acids and amino acids. Four metabolites were identified at level 2 when they matched the database (spectrum reverse match greater than 80% and retention index deviation lower than 1%). Twenty-two compounds were also detected with a level 3 confidence (spectrum reverse match greater than 80% and retention index greater than 1%), and seven compounds matched no database (level 4).

### 2.4. Application to the Comparison of Several Solanaceae Species

**Figure 2:**
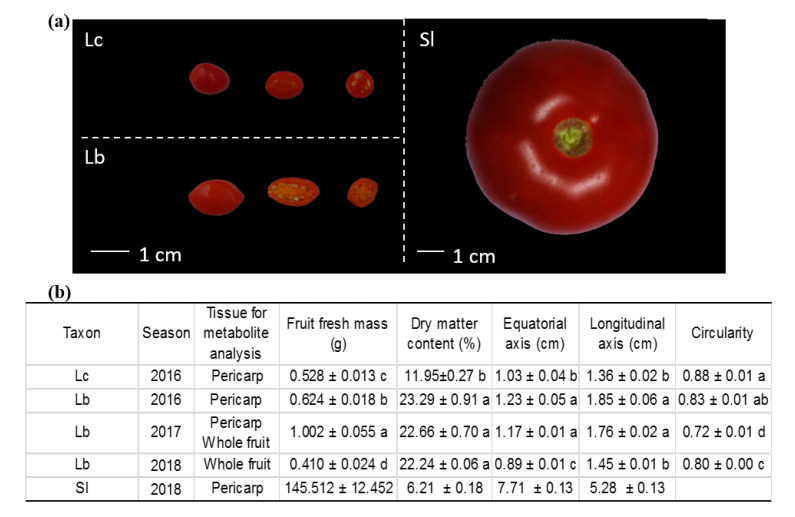
(**a**) Variations in morphological traits of 2 goji (*Lycium chinense* (Lc) and *Lycium barbarum* (Lb)) and tomato fruits *Solanum lycopersicum* (Sl). (**b**) Data on fruit harvest, fresh mass, dry matter content, size and circularity. Data are means ± standard errors (*n* > 4). Different letters indicate significant differences between Lc 2016, Lb 2016, 2017 and 2018 at α = 0.05. Accessions present significant variations in dry matter contents, Lb showing the highest values.

The methods were applied to analyze the metabolic profiles of several sets of goji and tomato fruits, namely Lb collected in 2016, 2017 and 2018; Lc collected in 2016; and Sl collected in 2018 (Figure 2).

**Figure 3:**
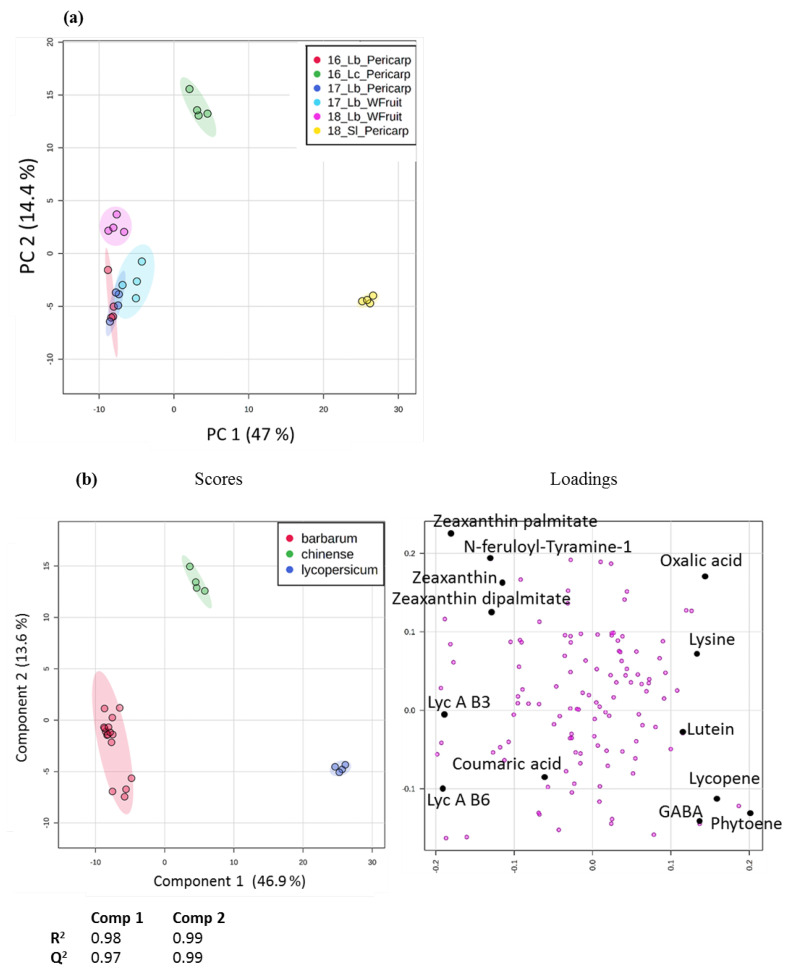
Multivariate analysis of the whole dataset, including 126 metabolites (peak intensities normalized by sample dry weight, log transformed and Pareto scaled) analyzed in 24 samples. (**a**) Score plot of principal component analysis (PCA) on 3 accessions: *Lycium barbarum* (Lb, in blue, pale blue, pink or dark blue on the score plot); *Lycium chinense* (Lc, in green); and *Solanum lycopersicum* (Sl, in yellow). The percentage of variance captured is indicated on each axis. Samples are labeled as follows: Year_Accession_Tissue (Wfruit = whole fruits). (**b**) Partial least-squares discriminant analysis (PLS-DA), R2 and Q2 are detailed below, and some of the most discriminant metabolites are labelled on the loadings plot.

A total of 59 secondary metabolites (13 carotenoids and 46 phenolic compounds) and 67 primary metabolites were profiled. A PCA performed on the whole dataset (Figure 3a) indicated that the main source of variation (47%) was associated with the genus *Lycopersicum* vs. *Lycium*, and that the second source of variation (14.4%) was associated with the *Lycium* species. Minor sources of variation appeared among Lb samples, associated with either the tissue sampling (16 phenolic compounds, Table S5) or the year of harvest (25 metabolites, including stress related branched amino acids, lycibarbarphenylpropanoids and remarkably no carotenoids, excepted for the putative auroxanthin myristate (Table S6)). A PLS-DA performed on the same data displayed good model performance with very high values of the test parameters R²Y and Q²Y (Figure 3b). It gave similar results as the PCA, the loadings plot revealing higher levels of lycopene in Sl, lycibarbarphenylpropanoids in Lb and zeaxanthin esters or phenolamides in Lc.

**Figure 4:**
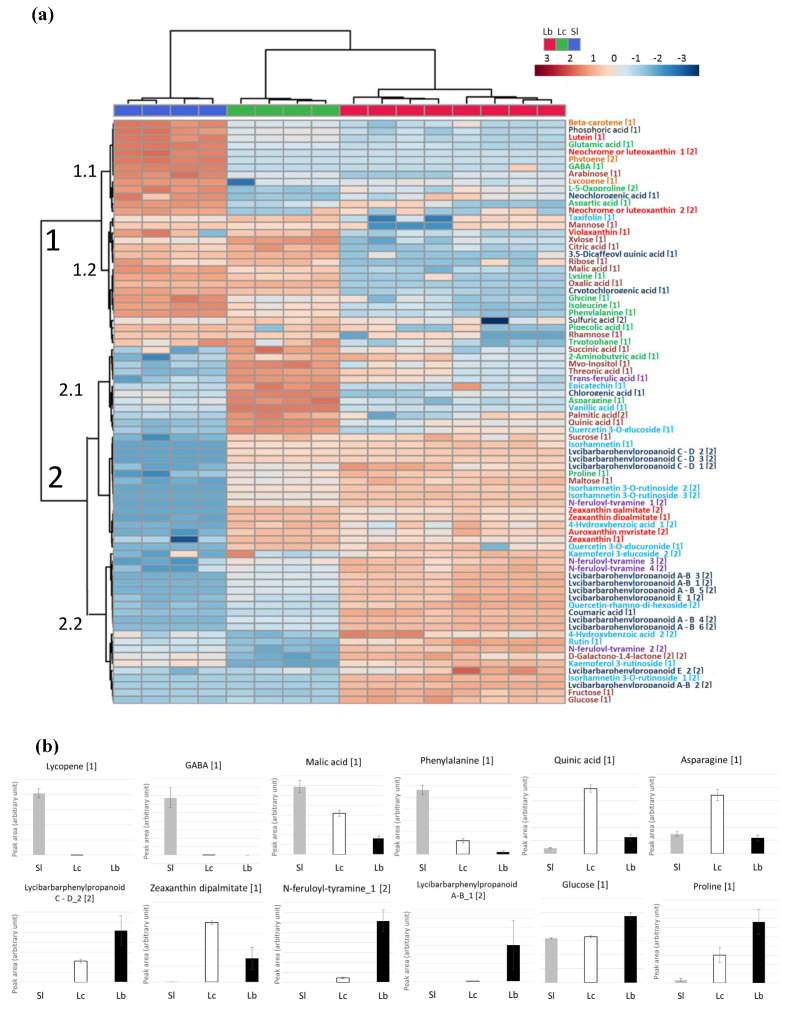
Metabolites discriminating the genotypes. (**a**) Heat map showing the expression (from blue for low content to red for high content) of the 80 relevant metabolites (Mann–Whitney *p* < 0.05 and identification confidence levels 1 and 2) in the 24 samples presented in rows. The dataset was composed of Log transformed and Pareto scaled values. The color code used for the accessions is blue for Sl, green for Lc and red for Lb. The metabolites appear in rows with a color code according to their chemical class: xanthophylls and derivatives in red; carotenes in orange; hydroxycinnamic acids and derivatives in deep blue; hydroxybenzoic acids, flavonols and derivatives in light blue; ferulic acid derivatives in purple; amino acids in green; organic acids and carbohydrates in brown. The identification confidence level is indicated between brackets after the compound name. The two classifications (accessions in columns and metabolites in rows) are based on Euclidian distances and Ward algorithm. (**b**) Mean and standard deviation bar plots of remarkable genotypic markers, ordinates are in arbitrary units.

These observations were best represented by a heatmap of hierarchical clustering on the same samples, based on metabolites identified at level 1 or 2 that exhibit significant differences between genotypes (Mann–Whitney U-test, *p* < 0.05 (Figure 4)). The metabolites formed four main clusters. The cluster 1.1 grouped the Sl markers, lycopene and carotene, more abundant in tomato fruits, but also lutein, or the amino acids GABA and its precursor glutamate. The second cluster, 1.2, contained markers found both in Sl and Lc, namely the major fruit organic acids citrate and malate and the aromatic amino acids phenylalanine and tryptophane. Cluster 2.1 was characteristic of *Lycium* metabolites, with lycibarbarphenylpropanoids C-D, sucrose or zeaxanthin and its esters, it was also worse, noting a set of compounds more specific to Lc: chlorogenic acid, asparagine, vanillic acid and quinic acid. The last cluster, 2.2, involved Lb markers such as lycibarbarphenylpropanoids A-B, coumaric acid, most feruloyl tyramine isomers and the sugars fructose and glucose.

The composition of goji fruits, especially in secondary metabolites, has been characterized by numerous authors [1,11,19,34,36,38,40]. However, the difference between Lc and Lb has not been well established yet [12,14,41,42]. According to Potterat [12], the two species presented similar accumulation of carotenoids, with zeaxanthin dipalmitate being the main component. Our study on the two species, grown in the same field, during the same season, showed that Lc presented larger amounts of free and esterified forms of zeaxanthin than Lb (more than +100%). Our analysis revealed additional metabolic markers of Lc related to the phenylpropanoid pathway. This accession accumulated vanillic acid as well as quinic acid and its hydroxycinnamate conjugates (chlorogenic acids and 3,5-dicaffeoylquinic acid), while Lb accumulated higher amounts of the precursor coumaric acid and flavonoids, such as kaempferol glucosides. Another interesting difference concerns the carbon balance; higher amounts of organic acids were found in Lc, while fructose and glucose levels were higher in Lb. Similarly, the nitrogen metabolism was contrasted between the two species, since Lb displayed much lower content in most amino acids.

The genus differences probably reflect different regulations of the carotenoid pathways and the presence of specialized metabolism in *Lycium* with the biosynthesis of lycibarbarphenylpropanoids. The amino acids phenylalanine and lysine and the secondary metabolites lycopene, β-carotene, lutein and cryptochlorogenic acid are commonly found in tomato fruits [43,44,45,46]. In comparison to Sl, our results suggest that the phenylalanine and shikimate biosynthetic pathways supplied more aromatic amino acids for phenylpropanoid metabolism in *Lycium* species than in the Sl. Moreover, other patterns, such as higher amounts of GABA in Sl or higher proline in *Lycium*, suggest possible contrasted stress response mechanisms in both genera.

## 3. Materials and Methods

### 3.1. Plant Materials

The *Lycium* “FPW07” and “Crimson Star” accessions were collected from a commercial orchard in the south of France, near Revel (43°46′ N 2° E), during three seasons from 2016 to 2018 (Figure S5). Both were given as *Lycium barbarum* (Lb); however, the phenotype of “Crimson Star” was reminiscent of *Lycium chinense* (Lc), and thus the amplification refractory mutation system (ARMS), recently described by Wetters et al. [14], was used to identify the genotypes. Briefly, PCRs with the universal chloroplastic psbA and TrnH primers combined with the LB_265T_fw or LC_265T_fw diagnostic primers allowed for the discrimination of the two related species and revealed that the (maternal) genome of the “FPW07” accession was Lb, whereas the accession provided as “Crimson Star” was Lc.

All plants were two-year-olds and were subjected to identical and commercial practices. Fruits were harvested at maturity 35 days after anthesis (DAA). Four independent samples of at least 10 fruits were collected on 4 plants for each species and season. Climatic data and collection dates during the three seasons are detailed in Figure S5. All fruits were characterized with the following indicators: fruit fresh and dry mass, fruit size and fruit circularity. The dry matter content was determined by weighing 3 g of fruit pericarp pieces before and after drying at 70 °C. Fruit size and fruit circularity were determined by image analysis using ImageJ software (https://imagej.nih.gov/ij/). In order to make comparisons with tomato (*Solanum lycopersicum*), the “Levovil” cultivar (normal round tomato) was grown under a glasshouse near Avignon (43°54′ N 4°52′ E), France, in 2018. Ten plants were grown in 9 L pots filled with compost (Substrate 4, Klasmann, Champety, France). Four samples of four fruits were collected at fruit maturity (55 DAA) on four plants. As for goji fruits, fruit fresh and dry mass and fruit size were determined; these data are summarized in Figure 2.

### 3.2. Metabolite Extraction

All chemical and metabolite standards were purchased from either Extrasynthese (Genay, France), Sigma-Aldrich (Steinheim, Germany) or CaroteNature (Ostermundigen, Switzerland). All solvents and reagents were analytical or LC/MS grade. Ultrapure water was used throughout the metabolite analysis.

Before extraction, fruit pericarps were separated from seeds (goji fruits) or placenta and seeds (tomato fruits), ground into fine powders in liquid nitrogen and stored at –80 °C until metabolite analysis. In 2017, comparison of metabolite profiles of goji pericarps and goji whole fruits showed very little difference; consequently, in 2018, only whole goji fruits were analyzed. A fraction of fruit powder was freeze-dried (Vacuum Freeze Dryer, Cryonext, Saint-Aunès, France) for the analysis of primary metabolites and phenolic compounds.

#### 3.2.1. Carotenoids

Carotenoid extraction was carried out from 50 mg of fresh fruit powder combined with 600 µL of ethanol (EtOH):hexane 2:1 (by volume) with butylated hydroxytoluene (BHT) (0.2%; *w*:*v*) and 50 mg/mL of CaCO_3_. Samples were immediately homogenized using a vortex for 20 s and a rotating wheel during 10 min at 30 rpm, in a dark room at ambient temperature. The extraction was completed by the sequential addition of 400 µL of ultrapure water and 800 µL of hexane with 0.1% (*w*:*v*) BHT, each addition being followed by a 20 s vortex shaking. The extracts were then centrifuged for 10 min at 16,100 rcf at 4 °C, and 800 µL of the upper phase were collected. The extraction was repeated by adding 800 µL of hexane with 0.1% (*w*:*v*) BHT, shaking for 20 s with a vortex and 10 min with a rotating wheel at 30 rpm in a dark room at ambient temperature. After centrifugation for 10 min at 16,100 rcf at 4 °C, 800 µL of the upper phase was collected. The extracts were combined, evaporated to dryness under a stream of nitrogen and then stored at –20 °C for a maximum of 15 days. Before injection, the dried extract was dissolved in 300 µL of methyl-tert-butyl ether (MTBE) and 600 µL of EtOH, filtered through a PTFE filter (Millex LG, PTFE from aqueous and organic, 0.2 µm, 4 mm, Millipore, Burlington, NC, USA) and poured in an amber vial for injection.

#### 3.2.2. Phenolic Compounds

Phenolics were extracted from 30 mg of dry goji or tomato fruits with 1.44 mL of methanol (MeOH) acidified with formic acid (5%; *v*/*v*) to break down membranes and solubilize compounds by decomplexion and protonation [37]. The extract was homogenized with a thermomixer (Eppendorf ThermoMixer C, Eppendorf AG 22331 Hamburg, Germany) for 10 min at 900 rpm at 70 °C, then 0.36 mL of ultrapure water was added before heating at 70 °C for 10 min at 900 rpm and centrifugation at 9300 rcf for 10 min at 10 °C. The supernatant was filtered through a 0.2 µm membrane filter and stored at –20 °C until analysis.

#### 3.2.3. Primary Metabolites

Primary metabolites were extracted from 5 mg of dry fruits with 1.44 mL of MeOH supplemented with adonitol (100 µM) as internal standard. Samples were homogenized with a thermomixer at 70 °C for 10 min at 900 rpm. Extracts were diluted with 0.36 mL of ultrapure water and homogenized again at 40 °C and 900 rpm for 10 min. Samples were then centrifuged at 16,200 rcf for 10 min at 4 °C. The supernatant was filtered through a 0.2 µm membrane filter and stored at –20 °C. Then, 50 µL of extract were transferred in a 2 mL glass vial and evaporated with a vacuum concentrator (Genevac miVac Duo Concentrator, SP Scientific, pswich, Suffolk, UK) at 40 °C for 12 h to dryness.

### 3.3. Metabolic Profiling by UPLC–DAD–ESI–TQ and GC–EI–TOF

#### 3.3.1. UPLC–DAD–ESI–TQ Analysis

The carotenoid analysis was carried out on a LC–MS equipped with a diode array detector (DAD) and a triple quadrupole mass spectrometer TQ–XS XEVO hyphenated to an Acquity UPLC Class I (Waters, Milford, MA, USA). The whole system was controlled by MassLynx version 4.2 (Waters, Milford, MA, USA). Chromatographic separation was achieved on a BEH C18 polymer column, 1.7 µm 2.1 × 50 mm, equipped with a guard column (Waters, USA), kept at 55 °C. The mobile phases consisted of acetonitrile/water (1:1, *v*/*v*) (eluent A) and isopropanol (eluent B), both containing 0.1% (*v*:*v*) formic acid and buffered with 10 mM ammonium formate to preserve carotenoids, according to de Quiros and Costa [23]. The carotenoids were eluted with the following gradient, initial condition: 0.2 mL/min, 65% A; down to 45.7% A in 4 min; then down to 36.1% A in 0.5 min at 0.3 mL/min; isocratic elution for 3.5 min and down to 0% A in 4 min; then the flow rate was decreased to 0.2 mL/min and kept for 1.5 min. A cleaning method was performed before returning to initial conditions: 3 consecutive injections of 10 µL of a blank solution (MTBE:EtOH, 1:2, by volume) using the following elution gradient at 0.2 mL/min: 0.6 min with 100% B, then back to the initial conditions 65% A in 3 min. The total run (sample run + cleaning method) was achieved in 18.3 min. The sampler operated at 20 °C. The DAD acquisition ranged from 270 to 600 nm in steps of 1.2 nm. The mass spectrometer was equipped with an ESI source at 150 °C in positive ion mode with a 2.5 kV capillary voltage, 450 °C desolvation temperature, 800 L/H desolvation gas flow and 150 L/H scanning cone flow. Mass acquisitions were performed in full scan mode (100–1200 uma) and MRM mode. To quantify carotenoids in goji fruits, two injections of 1 µL were performed: the first one consisted of a concentrated extract and the second of a diluted extract at 1/10 in MTBE/EtOH 1:2 (*v*/*v*). Linear external calibrations with available standards were used to quantify carotenoids. The concentrations of the standard solutions were determined prior to injection using a spectrophotometer (Infinite 200 option mono cuvette, Tecan Austria GmbH 5082 Grödig, Austria) and specific molar absorption coefficients as described by Britton [21] or provider Extra synthese (Table S7). Then a fraction of each standard was mixed, evaporated to dryness under a stream of nitrogen and dissolved in 300 µL MTBE and 600 µL EtOH just before injection.

The phenolic compounds analysis was carried out on the same equipment. There is a great variability in phenolics extraction protocols. It is classically carried out with a water/alcohol mixture. Comparisons of different extraction solvents, durations and temperatures have been reported [31,37,47,48]. Increases in temperature increase the solubility of the phenolic compounds, allowing for shorter extraction times, likely to reduce the decomposition of phenolic compounds [37]. These authors recommend an extraction of 162 min at 45 °C, whereas Lu et al. [47] note a degradation as soon as 40 min at 30 °C and thus recommend an extraction of 40 min at 50 °C to obtain the best yield. Wang et al. [49] even go up to 90 °C for 2 h. We chose a two-step extraction with a final solvent composition of MeOH:water:formic acid (80:20:4, *v*/*v*/*v*) for 10 min at a temperature of 70 °C. Two columns were tested: CSH C18 from Waters and F5 PFP from Phenomenex. Retention and peak resolution was found to be greater on the F5 PFP column (Kinetex F5, 1.7 µm 2.1 × 150 mm fitted to a Security Guard Ultra Cartridges UHPLC F5 2.1 mm ID guard column, Phenomenex, Torrance, CA, USA). The oven was set to 40 °C, and the mobile phases consisted of water (eluent A) and MeOH (eluent B), both containing 0.1% (*v*:*v*) formic acid. The flow rate was 0.25 mL/min and the gradient as follows: 99.5% A maintained during 1.5 min, down to 75% A in 3 min, to 20% in 4.5 min and to 0% in 3 min maintained for 2 min, then back to 99.5% A in 1 min. The total run was achieved in 17 min. The sampler operated at 10 °C to limit compound degradation. The DAD acquisition ranged from 200 to 800 nm in steps of 1.2 nm. The mass spectrometer was equipped with an electrospray source (ESI) at 150 °C in negative ion mode with a 2.5 kV capillary voltage, 550 °C desolvation temperature, 1000 L/H desolvation gas flow and 150 L/H scanning cone flow. Mass acquisitions were performed in scan mode (100–1200 uma) and MRM mode. To quantify phenolic compounds, 2 different volumes were used depending on sample concentration (1 or 3 µL). Linear external calibrations with available standards were used to quantify phenolic compounds. Between every sample injection, 10 µL of a blank solution (MeOH/water, 4:1, by volume) was injected to clean the injection system.

#### 3.3.2. GC–EI–TOF Analysis

The method was adapted from Roessner et al. [15]. Samples were derivatized online before injection with a MultiPurpose Sampler (Gerstel MPS, CTC Analytics AG, Mülheim an der Ruhr, Switzerland): dried extract were incubated in 50 µL of a pyridine solution containing 20 mg/mL of methoxyamine hydrochloride under constant shaking at 900 rpm and 80 °C for 90 min. Then, 80 µL of BSTFA containing a mixture of 9 n-alcanes were added before heating for 30 min at 80 °C under constant shaking at 900 rpm. Data acquisition was performed with a gas chromatograph system (7890B GC, Agilent Technologies, Santa Clara, CA, USA) equipped with a capillary column (ZB-SemiVolatiles, 34.59 m, internal diameter 250 µm, film thickness 250 µm, Phenomenex, Torrance, USA) hyphenated to a TOF mass spectrometer (Pegasus BT, Leco, Saint Joseph, Benton Harbor, MI, USA). One microliter of sample was injected in split mode (1:50) at 230 °C. Helium was used as carrier gas at 0.6 mL/min. The initial oven temperature was kept at 70 °C for 1 min and then increased to 320 °C (9 °C/min) and maintained for 10 min. The *m/z* scan range was 70–600 with a cycle time of 20 scans/s. Source temperature and transfer line were set at 250 °C. The MultiPurpose Sampler was controlled by Maestro Version 1.4.40.1. Gerstel and gas chromatography system with mass spectrometer were controlled by ChromaTOF Version 5.20.38.0.54864 (LECO, Saint Joseph, MI, USA).

#### 3.3.3. Data Processing and Statistical Analysis

UPLC–DAD–ESI–TQ data were processed with the TargetLynx software (Waters, USA) to integrate peaks in MRM mode. Peak areas were normalized against the sample dry weight and the extraction and injection volumes. GC–EI–TOF data were deconvoluted with the LECO NTD software (LECO, USA), and peak annotation was achieved using a mass spectral library (Golm database, Nist 2014, Leco-fiehn rtx5). A specific extracted ion chromatogram (XIC) was chosen for each molecule for integration; then peak areas were normalized against the sample dry weight, the internal standard (adonitol) and the extraction volume.

The datasets obtained by UPLC–DAD–ESI–TQ or GC–EI–TOF comprised 13 carotenoids, 46 phenolic compounds and 67 primary metabolites, determined in 24 samples. Null values were replaced by an arbitrary value (0.01) that remained smaller than one tenth of the minimum value in the dataset. Principal component analysis (PCA), PLS-DA and heatmap clustering were performed on the data after log transformation and Pareto scaling with Metaboanalyst 4.0 [50]. Mann–Whitney U-tests were performed with Statistica (Statsoft).

A mixture of goji fruits representative of all the goji samples was extracted and used for the optimization and validation tests. Table S8 displays the parameters tested for each targeted metabolite class and according to five objectives, namely improved extraction yield, metabolite stability, chromatographic performances, mass specificity and quantitative validation. The quantitative validation included the determination of limit of quantification (LOQ), linearity, precision and matrix effect. LOQ was determined as the lowest concentration in the calibration curves of authentic standards showing a signal to noise ratio greater than 10.

## 4. Conclusions

We developed a set of UPLC–MS and GC–MS-based analytical methods for the reliable determination of metabolites in goji berries. The methods comprise optimized extraction processes and are suitable for semi-quantitative and quantitative analysis of the main central metabolites and secondary metabolite classes. An important achievement is the profiling of 13 carotenoids in 18 min that relies on the most common chromatographic devices and ionization source. The main limitations concerning detector saturation and compound stability have been overcome and a proof of concept has been established by comparing three genotypes. Specific markers of tomato and *Lycium* have been clearly exposed, but also for the first time to our knowledge, characteristic metabolic differences between Lb and Lc. Future studies aiming at crop genetic improvement could take advantage of this work to assess the nutritional value of goji berries or to monitor the impact of the environmental conditions on fruit quality.

## Supplementary Materials

The following are available online at https://www.mdpi.com/2218-1989/10/10/422/s1: Figure S1. DAD chromatograms of carotenoids mixture separated on a C18 UPLC column kept at 30 or 55 °C. Figure S2. Full scan positive ESI–MS mass spectra of carotenoids in standard solution and tomato/goji berry extracts. Figure S3: UPLC–ESI–DAD–TQ chromatograms of carotenoids. Figure S4. Phenolic compound extraction optimization and quantitative analysis. Figure S5. Daily minimum and maximum air temperature, rainfall and solar radiation monitored in field in 2016, 2017, and 2018. Table S1. List of compounds identified by UPLC–DAD–ESI–TQ. Table S2. Response linearity r2 calculated from standard mixture at 8 concentrations. Table S3. Precision of the method evaluated as the ESI peak areas coefficient of variation (%) from 15 injections. Table S4. List of compounds identified by GC–TOF. Table S5. Mann–Whitney U-Test for the comparison of Lb pericarp and whole fruit metabolic content (*p* < 0.05). Table S6. Mann–Whitney U-Test for the comparison of Lb pericarp metabolic content harvested in 2016 and 2017 (*p* < 0.05). Table S7. Properties of carotenoids. Table S8. List of the tests performed for method optimization, covering three metabolite classes.
