# Supplementary material for: Multi-Targeted Metabolic Profiling of Carotenoids, Phenolic Compounds and Primary Metabolites in Goji (Lycium spp.) Berry and Tomato (Solanum lycopersicum) Reveals Inter and Intra Genus Biomarkers
Source: Metabolites. 2020 Oct 21;10(10):422. doi: 10.3390/metabo10100422 (PMC7589643; doi:10.3390/metabo10100422)
Supplement: Supplementary file 1 [file metabolites-10-00422-s001.pptx]

## Slide 1
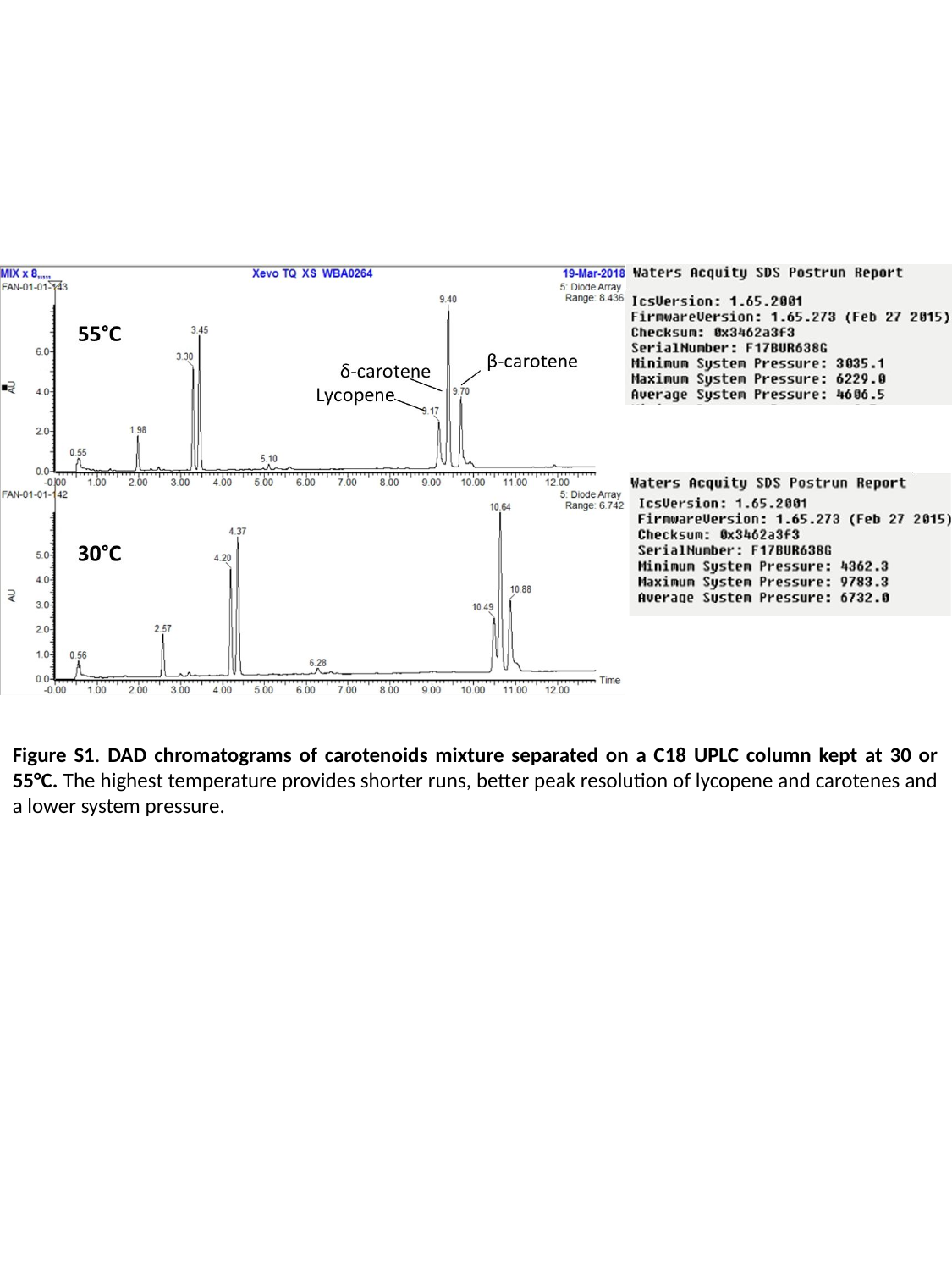

Figure S1. DAD chromatograms of carotenoids mixture separated on a C18 UPLC column kept at 30 or 55°C. The highest temperature provides shorter runs, better peak resolution of lycopene and carotenes and a lower system pressure.

## Slide 2
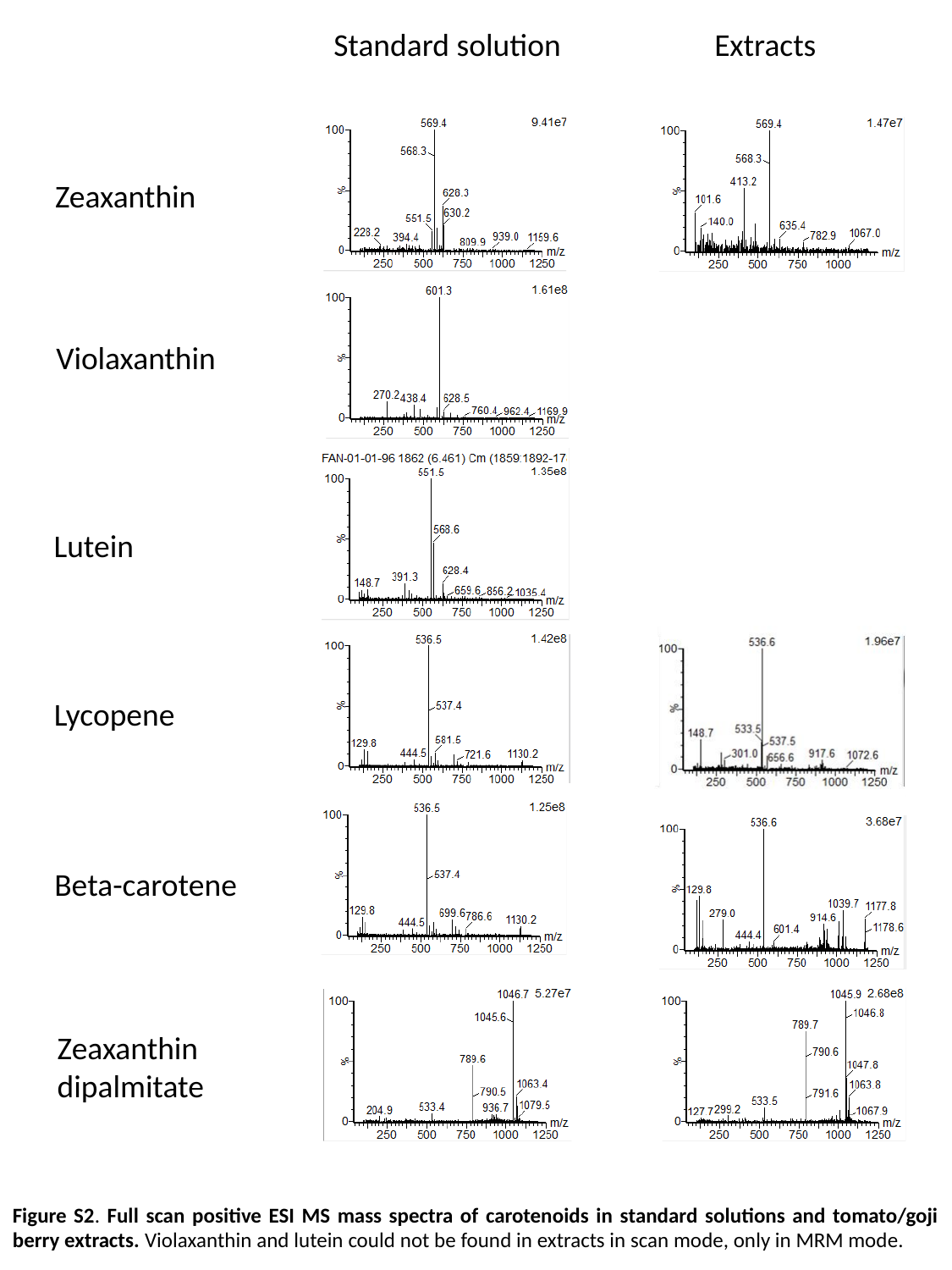

Standard solution		Extracts
Zeaxanthin
Violaxanthin
Lutein
Lycopene
Beta-carotene
Zeaxanthin dipalmitate
Figure S2. Full scan positive ESI MS mass spectra of carotenoids in standard solutions and tomato/goji berry extracts. Violaxanthin and lutein could not be found in extracts in scan mode, only in MRM mode.

## Slide 3
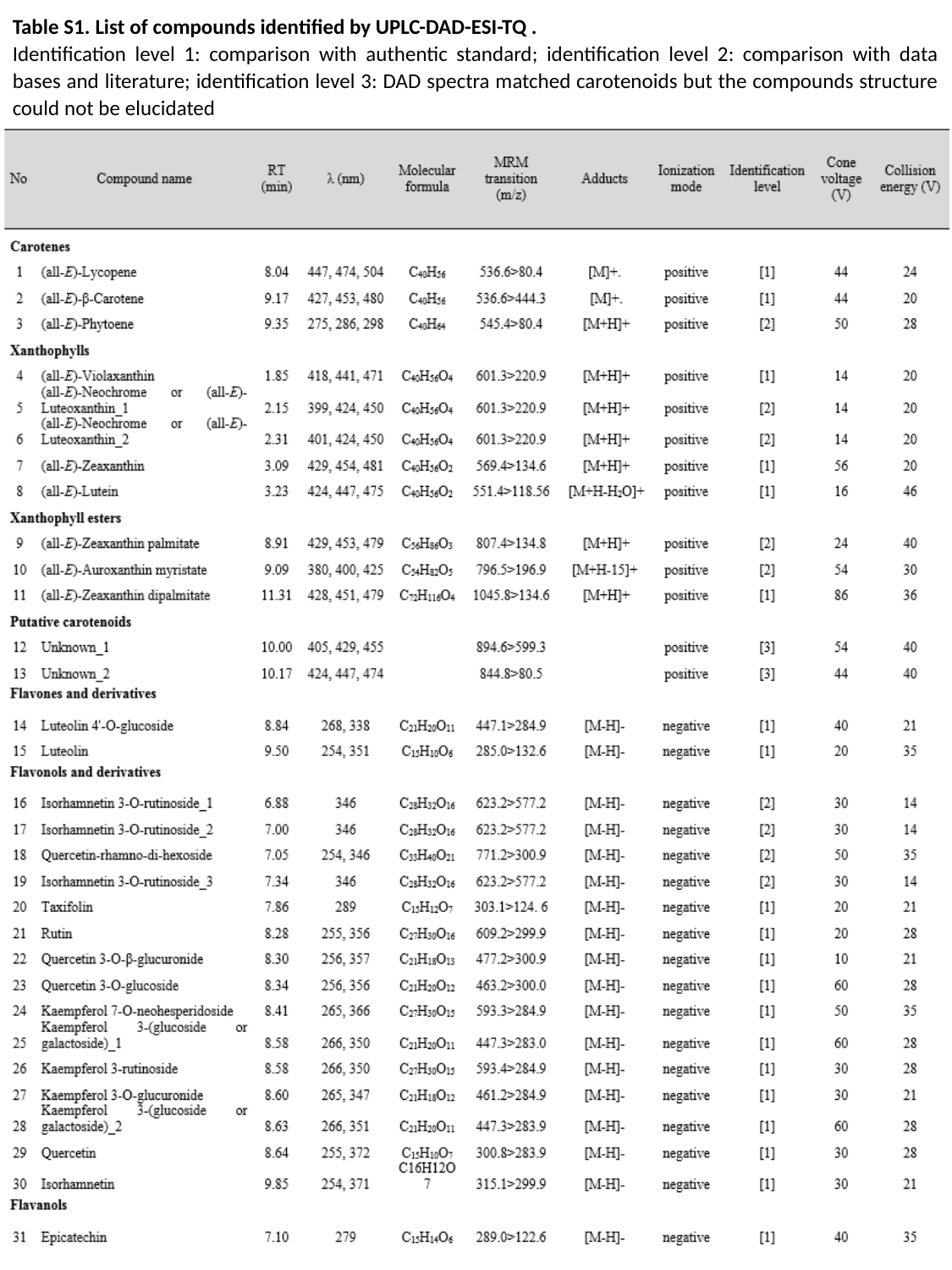

Table S1. List of compounds identified by UPLC-DAD-ESI-TQ .
Identification level 1: comparison with authentic standard; identification level 2: comparison with data bases and literature; identification level 3: DAD spectra matched carotenoids but the compounds structure could not be elucidated

## Slide 4
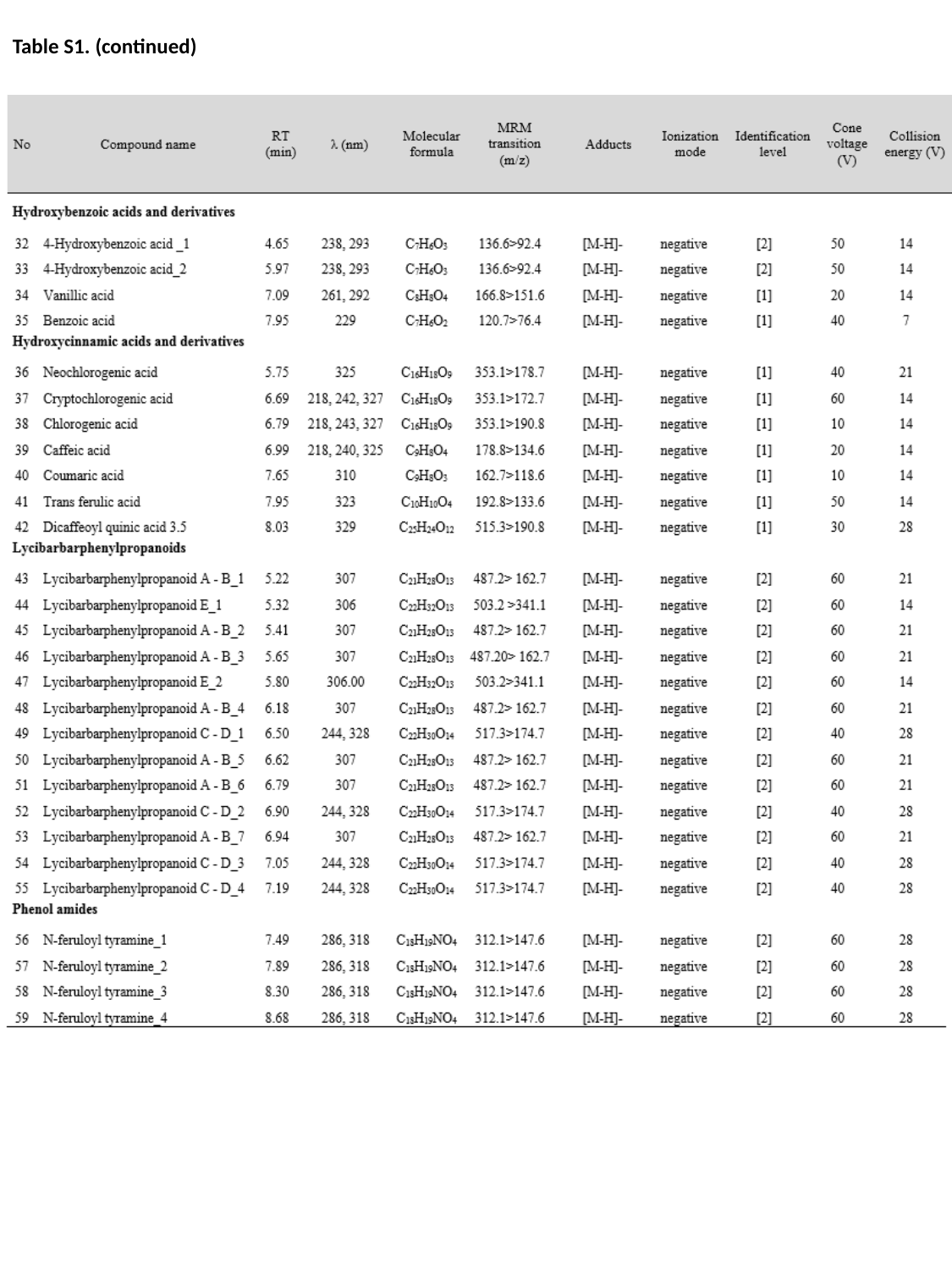

Table S1. (continued)

## Slide 5
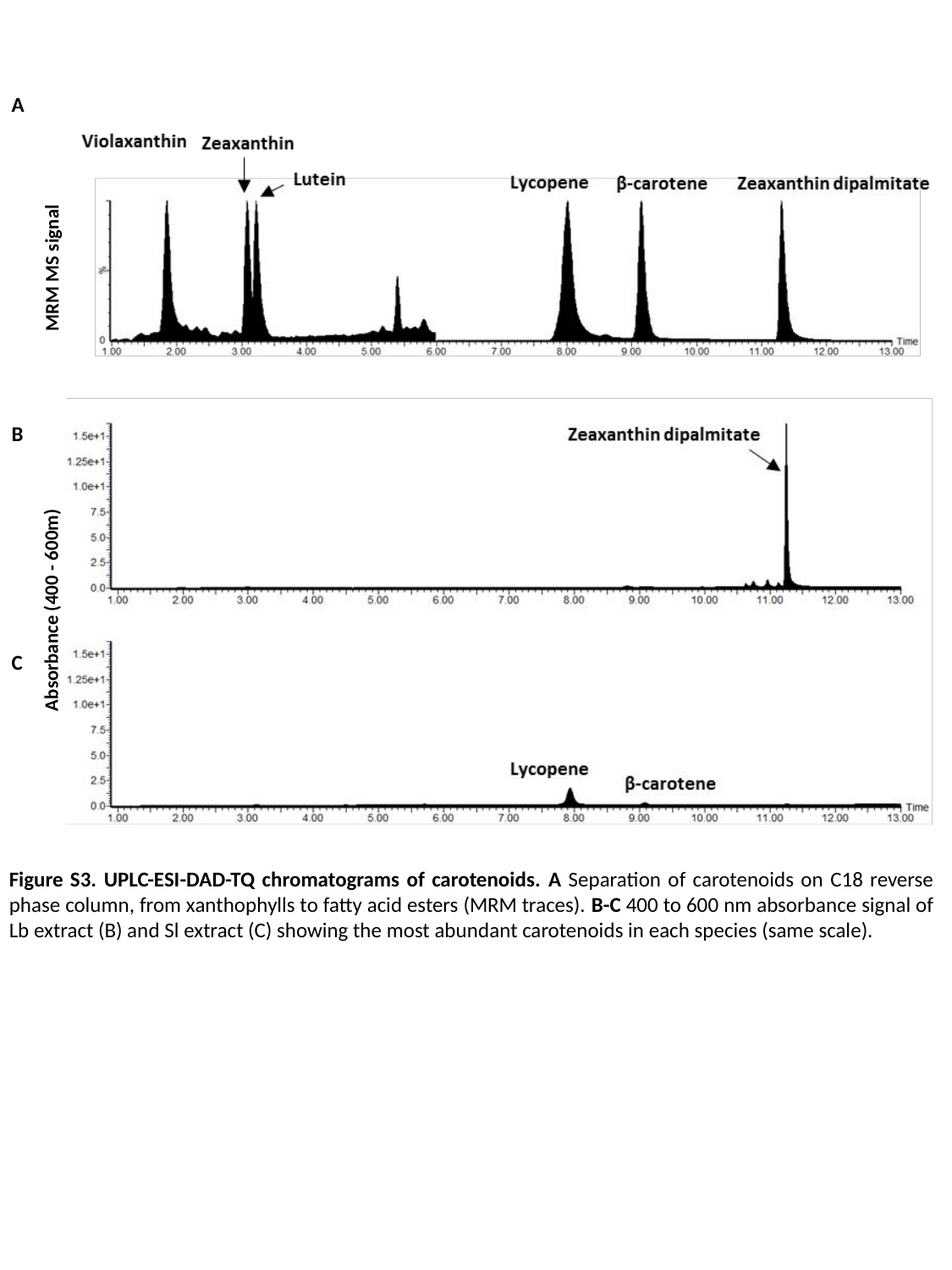

A
B
C
MRM MS signal
Absorbance (400 - 600m)
Figure S3. UPLC-ESI-DAD-TQ chromatograms of carotenoids. A Separation of carotenoids on C18 reverse phase column, from xanthophylls to fatty acid esters (MRM traces). B-C 400 to 600 nm absorbance signal of Lb extract (B) and Sl extract (C) showing the most abundant carotenoids in each species (same scale).

## Slide 6
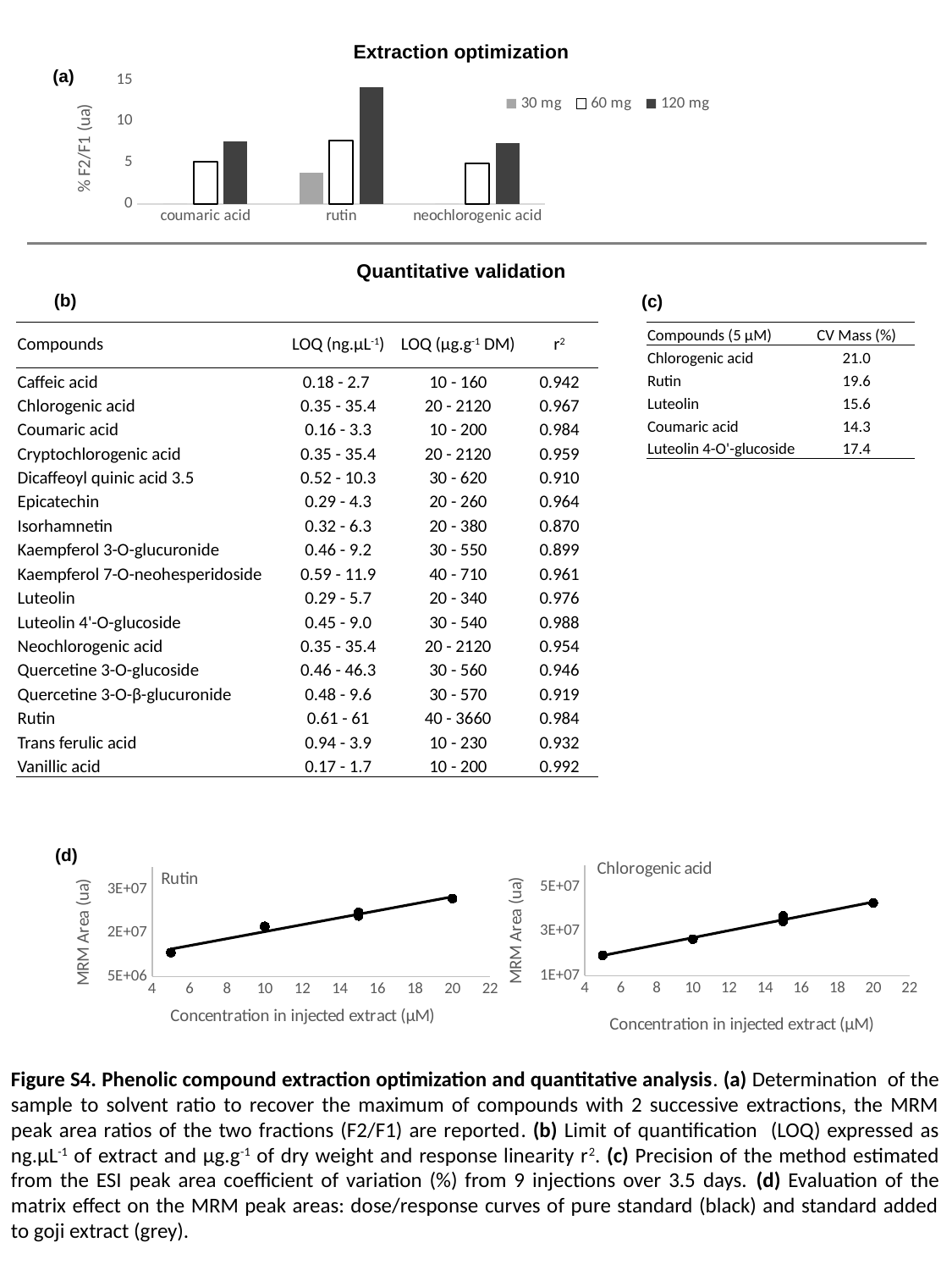

Extraction optimization
(a)
### Chart
| Category | 30 mg | 60 mg | 120 mg |
|---|---|---|---|
| coumaric acid | 0.0 | 5.060317522481505 | 7.586514051158772 |
| rutin | 3.7752691123406468 | 7.713349585054665 | 14.124246423237425 |
| neochlorogenic acid | 0.0 | 4.918909346903465 | 7.387966594554857 |Quantitative validation
(b)
(c)
| Compounds | LOQ (ng.µL-1) | LOQ (µg.g-1 DM) | r2 |
| --- | --- | --- | --- |
| Caffeic acid | 0.18 - 2.7 | 10 - 160 | 0.942 |
| Chlorogenic acid | 0.35 - 35.4 | 20 - 2120 | 0.967 |
| Coumaric acid | 0.16 - 3.3 | 10 - 200 | 0.984 |
| Cryptochlorogenic acid | 0.35 - 35.4 | 20 - 2120 | 0.959 |
| Dicaffeoyl quinic acid 3.5 | 0.52 - 10.3 | 30 - 620 | 0.910 |
| Epicatechin | 0.29 - 4.3 | 20 - 260 | 0.964 |
| Isorhamnetin | 0.32 - 6.3 | 20 - 380 | 0.870 |
| Kaempferol 3-O-glucuronide | 0.46 - 9.2 | 30 - 550 | 0.899 |
| Kaempferol 7-O-neohesperidoside | 0.59 - 11.9 | 40 - 710 | 0.961 |
| Luteolin | 0.29 - 5.7 | 20 - 340 | 0.976 |
| Luteolin 4'-O-glucoside | 0.45 - 9.0 | 30 - 540 | 0.988 |
| Neochlorogenic acid | 0.35 - 35.4 | 20 - 2120 | 0.954 |
| Quercetine 3-O-glucoside | 0.46 - 46.3 | 30 - 560 | 0.946 |
| Quercetine 3-O-β-glucuronide | 0.48 - 9.6 | 30 - 570 | 0.919 |
| Rutin | 0.61 - 61 | 40 - 3660 | 0.984 |
| Trans ferulic acid | 0.94 - 3.9 | 10 - 230 | 0.932 |
| Vanillic acid | 0.17 - 1.7 | 10 - 200 | 0.992 |
| Compounds (5 µM) | CV Mass (%) |
| --- | --- |
| Chlorogenic acid | 21.0 |
| Rutin | 19.6 |
| Luteolin | 15.6 |
| Coumaric acid | 14.3 |
| Luteolin 4-O'-glucoside | 17.4 |
### Chart: Chlorogenic acid
| Category | | |
|---|---|---|
### Chart: Rutin
| Category | | |
|---|---|---|(d)
Figure S4. Phenolic compound extraction optimization and quantitative analysis. (a) Determination of the sample to solvent ratio to recover the maximum of compounds with 2 successive extractions, the MRM peak area ratios of the two fractions (F2/F1) are reported. (b) Limit of quantification (LOQ) expressed as ng.µL-1 of extract and µg.g-1 of dry weight and response linearity r2. (c) Precision of the method estimated from the ESI peak area coefficient of variation (%) from 9 injections over 3.5 days. (d) Evaluation of the matrix effect on the MRM peak areas: dose/response curves of pure standard (black) and standard added to goji extract (grey).

## Slide 7
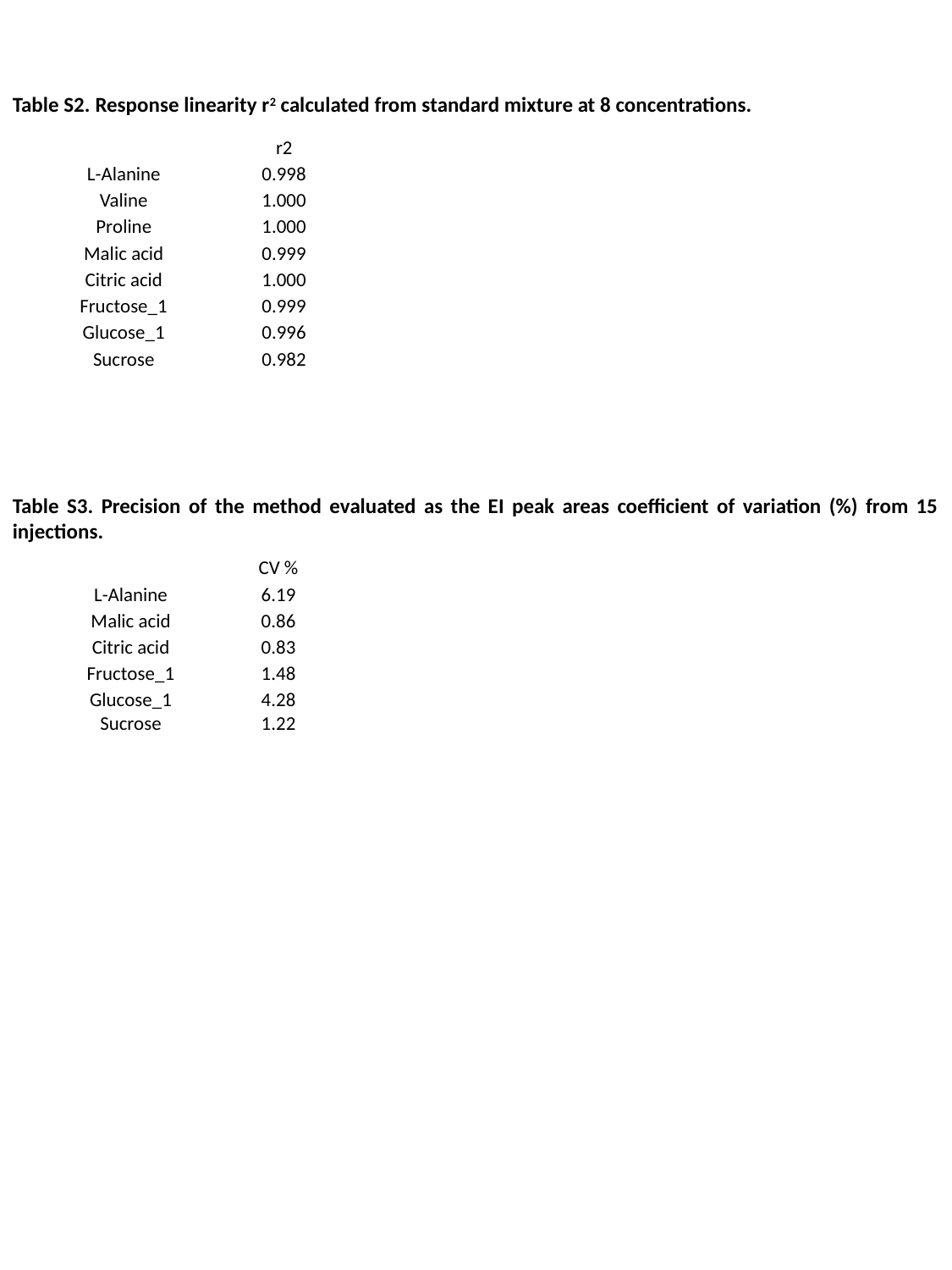

Table S2. Response linearity r2 calculated from standard mixture at 8 concentrations.
| | r2 |
| --- | --- |
| L-Alanine | 0.998 |
| Valine | 1.000 |
| Proline | 1.000 |
| Malic acid | 0.999 |
| Citric acid | 1.000 |
| Fructose\_1 | 0.999 |
| Glucose\_1 | 0.996 |
| Sucrose | 0.982 |
Table S3. Precision of the method evaluated as the EI peak areas coefficient of variation (%) from 15 injections.
| | CV % |
| --- | --- |
| L-Alanine | 6.19 |
| Malic acid | 0.86 |
| Citric acid | 0.83 |
| Fructose\_1 | 1.48 |
| Glucose\_1 | 4.28 |
| Sucrose | 1.22 |

## Slide 8
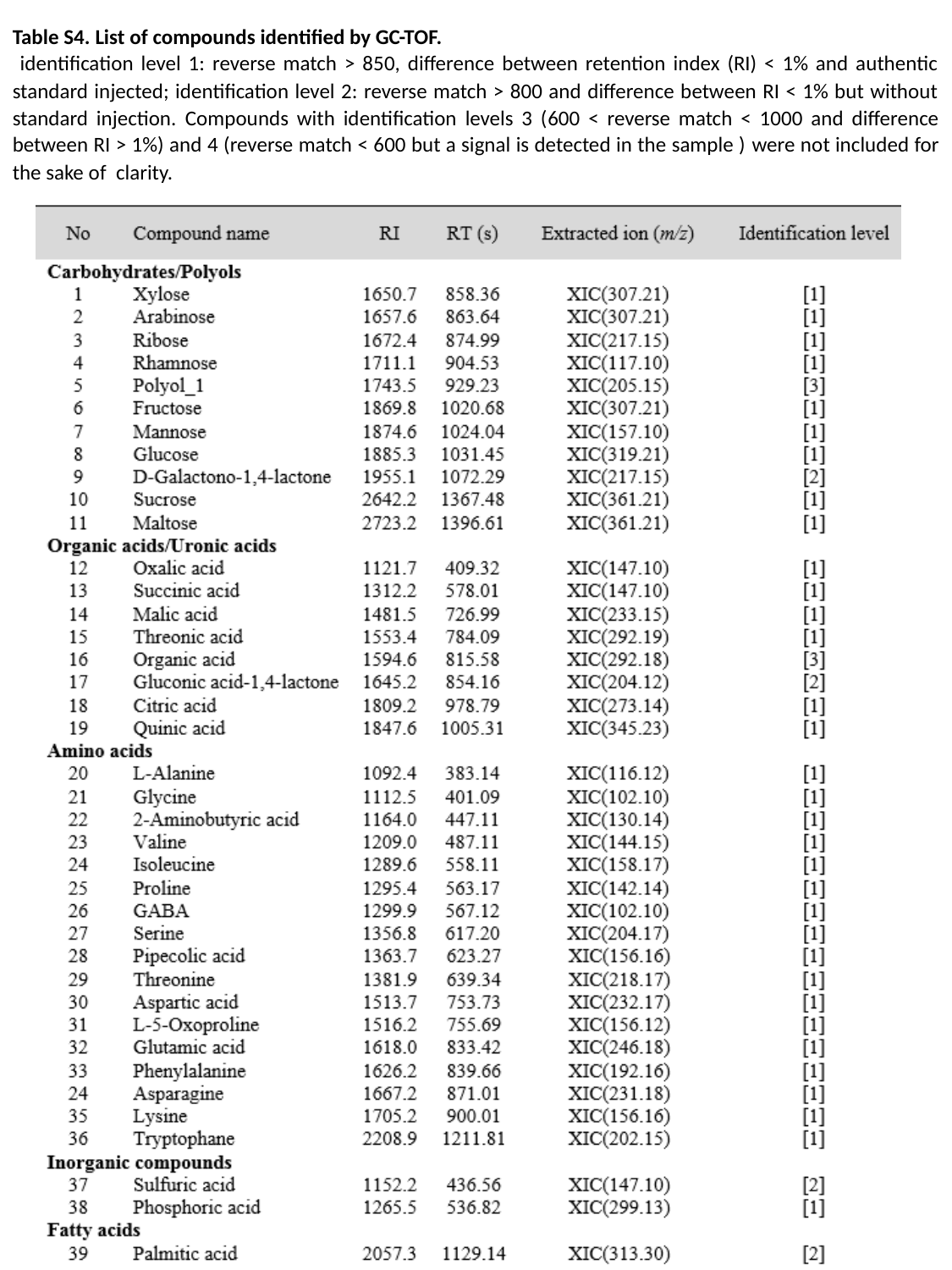

Table S4. List of compounds identified by GC-TOF.
 identification level 1: reverse match > 850, difference between retention index (RI) < 1% and authentic standard injected; identification level 2: reverse match > 800 and difference between RI < 1% but without standard injection. Compounds with identification levels 3 (600 < reverse match < 1000 and difference between RI > 1%) and 4 (reverse match < 600 but a signal is detected in the sample ) were not included for the sake of clarity.

## Slide 9
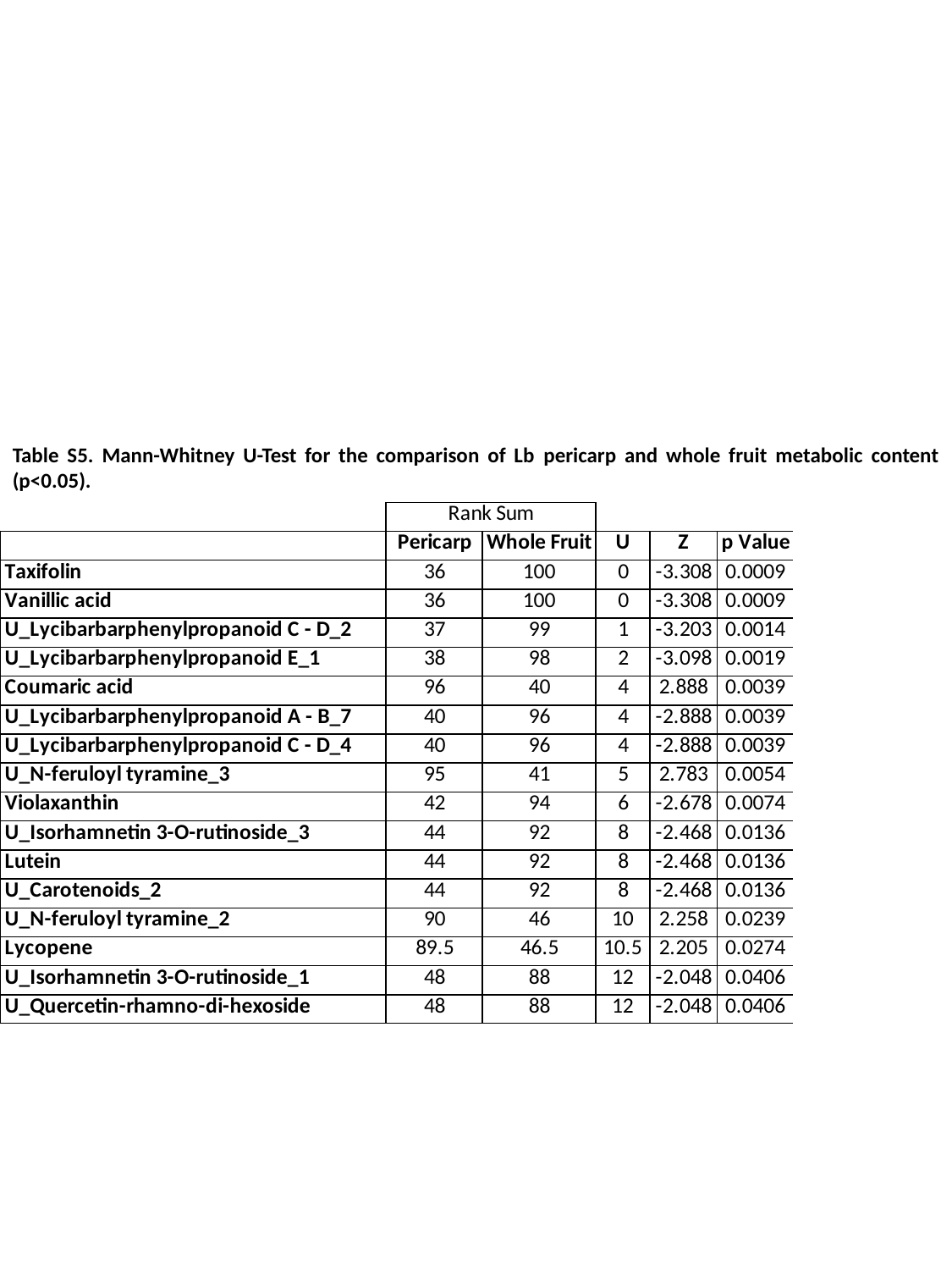

Table S5. Mann-Whitney U-Test for the comparison of Lb pericarp and whole fruit metabolic content (p<0.05).

## Slide 10
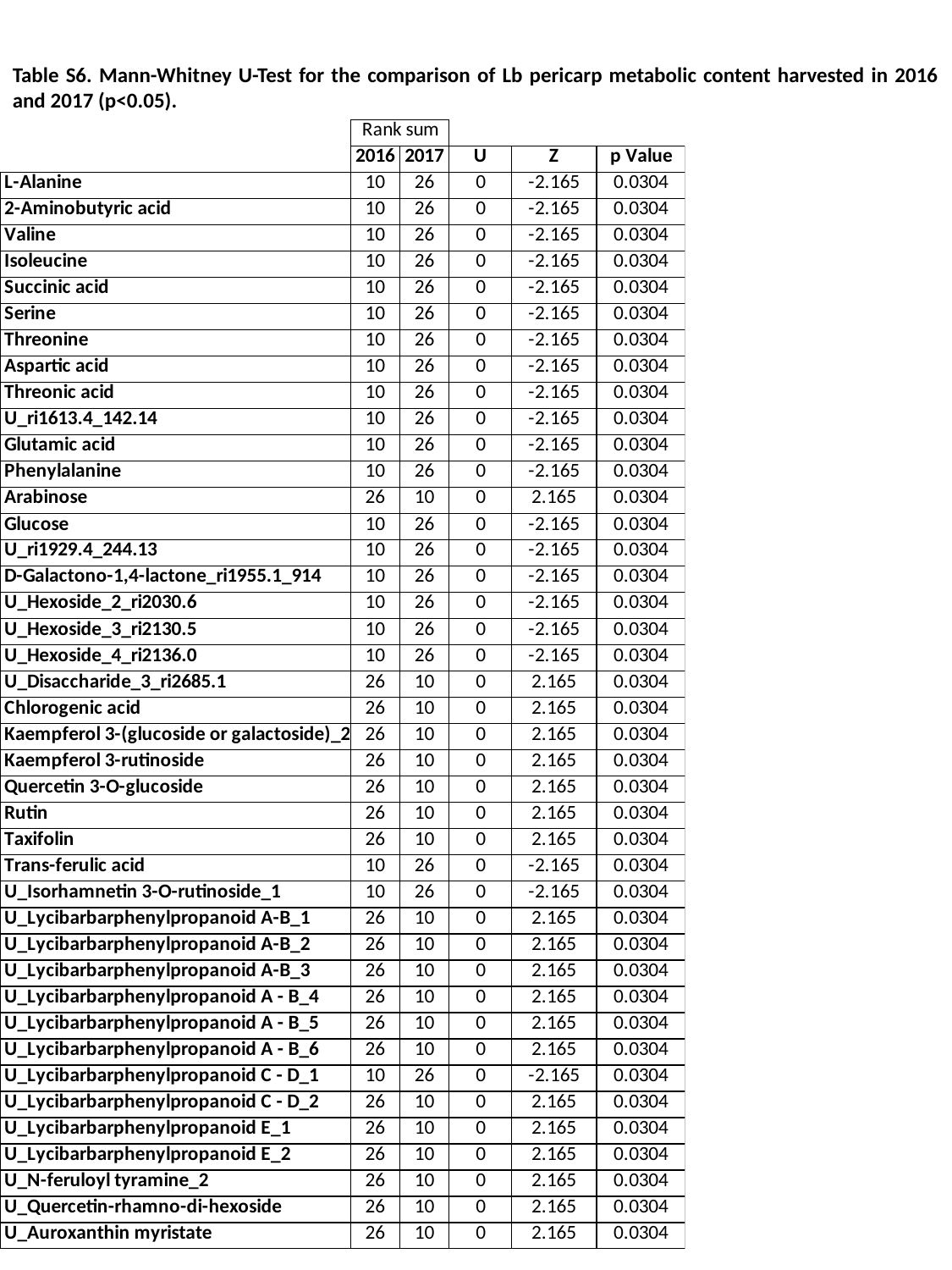

Table S6. Mann-Whitney U-Test for the comparison of Lb pericarp metabolic content harvested in 2016 and 2017 (p<0.05).

## Slide 11
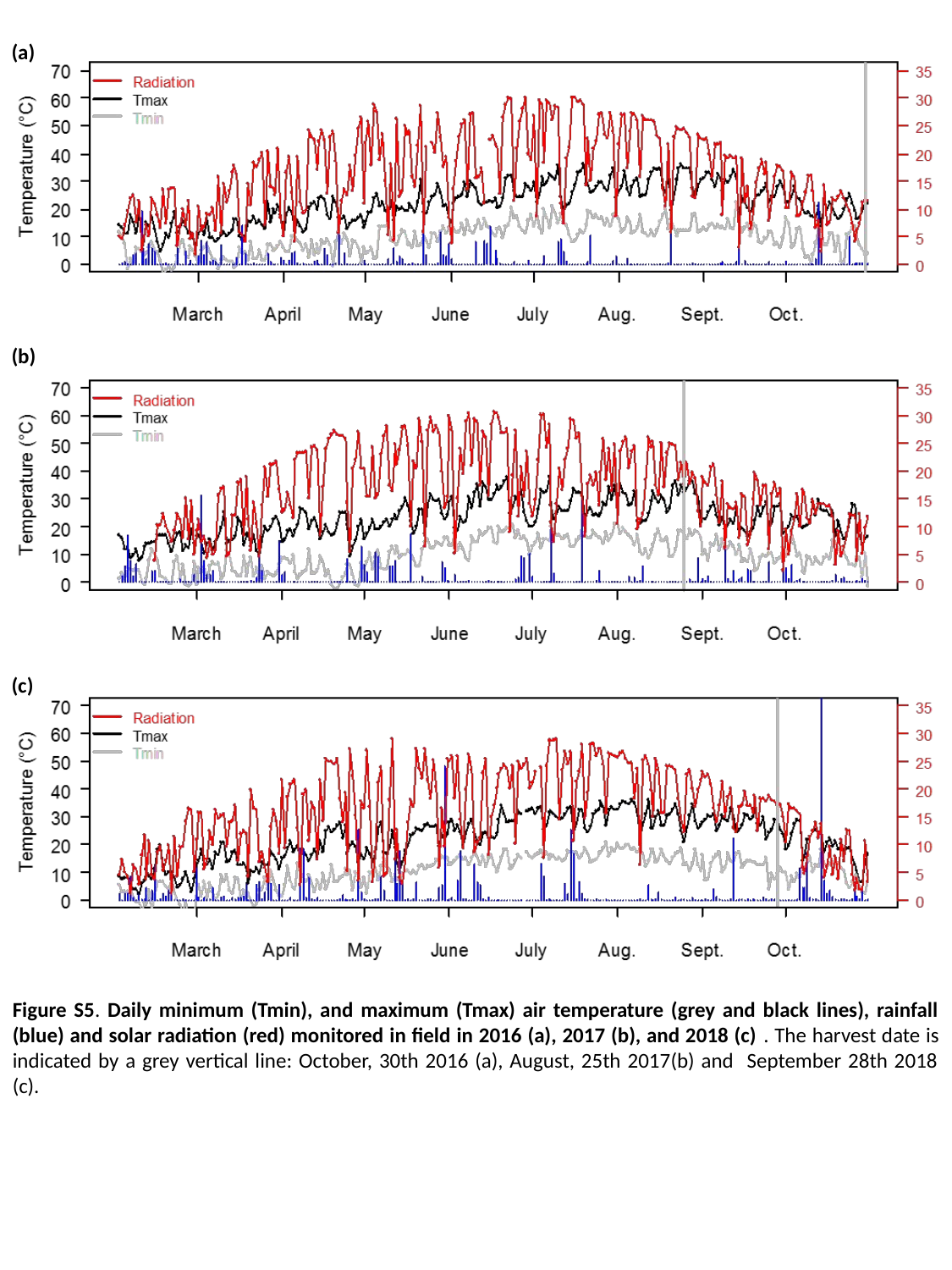

(a)
(b)
(c)
Figure S5. Daily minimum (Tmin), and maximum (Tmax) air temperature (grey and black lines), rainfall (blue) and solar radiation (red) monitored in field in 2016 (a), 2017 (b), and 2018 (c) . The harvest date is indicated by a grey vertical line: October, 30th 2016 (a), August, 25th 2017(b) and September 28th 2018 (c).

## Slide 12
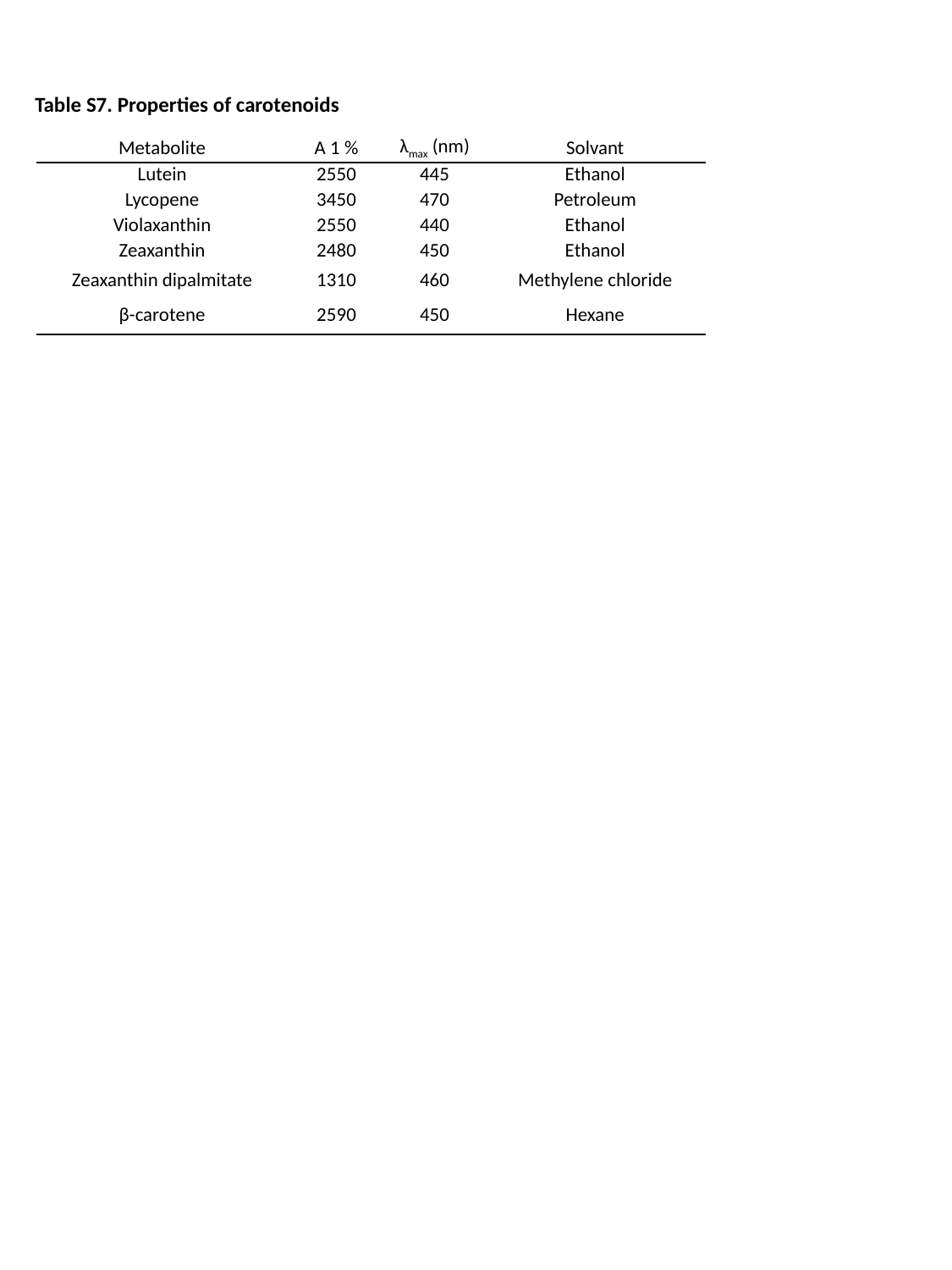

Table S7. Properties of carotenoids
| Metabolite | A 1 % | λmax (nm) | Solvant |
| --- | --- | --- | --- |
| Lutein | 2550 | 445 | Ethanol |
| Lycopene | 3450 | 470 | Petroleum |
| Violaxanthin | 2550 | 440 | Ethanol |
| Zeaxanthin | 2480 | 450 | Ethanol |
| Zeaxanthin dipalmitate | 1310 | 460 | Methylene chloride |
| β-carotene | 2590 | 450 | Hexane |

## Slide 13
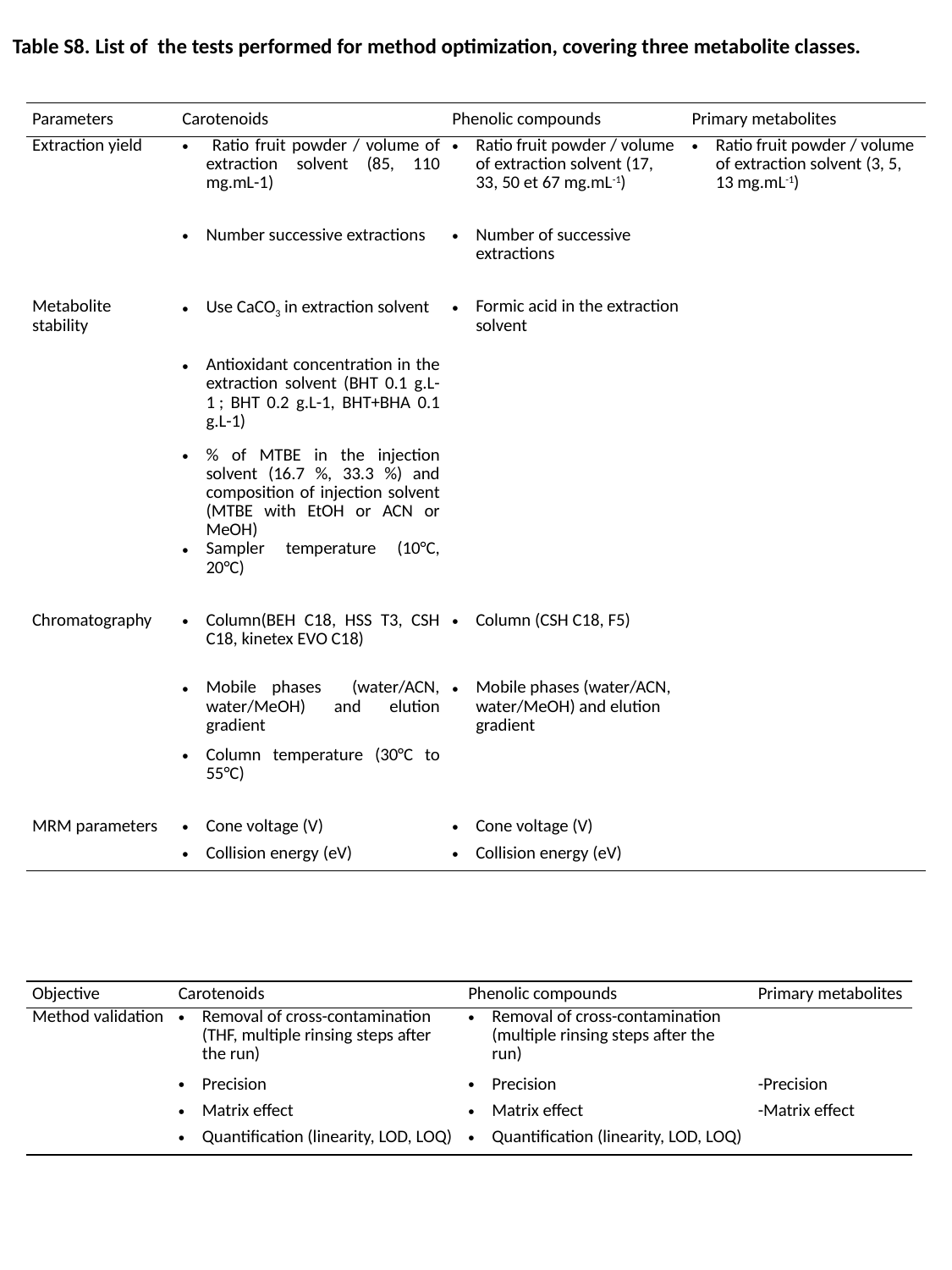

Table S8. List of the tests performed for method optimization, covering three metabolite classes.
| | | | |
| --- | --- | --- | --- |
| Parameters | Carotenoids | Phenolic compounds | Primary metabolites |
| Extraction yield | Ratio fruit powder / volume of extraction solvent (85, 110 mg.mL-1) | Ratio fruit powder / volume of extraction solvent (17, 33, 50 et 67 mg.mL-1) | Ratio fruit powder / volume of extraction solvent (3, 5, 13 mg.mL-1) |
| | Number successive extractions | Number of successive extractions | |
| | | | |
| Metabolite stability | Use CaCO3 in extraction solvent | Formic acid in the extraction solvent | |
| | Antioxidant concentration in the extraction solvent (BHT 0.1 g.L-1 ; BHT 0.2 g.L-1, BHT+BHA 0.1 g.L-1) | | |
| | % of MTBE in the injection solvent (16.7 %, 33.3 %) and composition of injection solvent (MTBE with EtOH or ACN or MeOH) | | |
| | Sampler temperature (10°C, 20°C) | | |
| | | | |
| Chromatography | Column(BEH C18, HSS T3, CSH C18, kinetex EVO C18) | Column (CSH C18, F5) | |
| | Mobile phases (water/ACN, water/MeOH) and elution gradient | Mobile phases (water/ACN, water/MeOH) and elution gradient | |
| | Column temperature (30°C to 55°C) | | |
| | | | |
| MRM parameters | Cone voltage (V) | Cone voltage (V) | |
| | Collision energy (eV) | Collision energy (eV) | |
| | | | |
| --- | --- | --- | --- |
| Objective | Carotenoids | Phenolic compounds | Primary metabolites |
| Method validation | Removal of cross-contamination (THF, multiple rinsing steps after the run) | Removal of cross-contamination (multiple rinsing steps after the run) | |
| | Precision | Precision | -Precision |
| | Matrix effect | Matrix effect | -Matrix effect |
| | Quantification (linearity, LOD, LOQ) | Quantification (linearity, LOD, LOQ) | |
